# Histological Grading and Prognosis in Breast Cancer

**DOI:** 10.1038/bjc.1957.43

**Published:** 1957-09

**Authors:** H. J. G. Bloom, W. W. Richardson

## Abstract

**Images:**


### HISTOLOGICAL GRADING AND PROGNOSIS IN BREAST CANCER

### A Study of 1409 Cases of which 359 have been Followed for 15 Years

H. J. G. BLOOM and W. W. RICHARDSON

*From the Meyerstein Institute of Radiotherapy and the Bland-Sutton Institute of Pathology of the Middlesex Hospital, London, W.1*

Received for publication July 29, 1957

THERE is still no general agreement as to the most suitable method of treating operable carcinoma of the breast. We believe that the difficultes in assessing the relative merits of greater and lesser surgical procedures, and the value of radiotherapy in these cases is largely due to the comparison of results in groups of patients which are not strictly comparable.

There is great variation in the progress of cases of breast cancer even in patients of the same age, with the same duration of symptoms, and with tumours of comparable clinical extent. Women with advanced disease and a long history may survive for many years following only limited treatment, whilst some patients who attend hospital early with what appears to be a localised growth, may die of metastases within twelve months of radical surgery and a full course of post-operative radiotherapy.

Have we a classification which takes into account the wide range of behaviour in carcinoma of the breast ? The practice of grouping patients according to a system of clinical staging is in general use at the present time, and although of considerable value does not take into full account the nature of the tumour itself. Thus whilst clinical staging provides a guide to the obvious extent of a tumour, it fails completely to indicate the likelihood of occult lymphatic and blood-born metastases being present in what appears to be an early case, nor the speed with which such metastases may develop.

An indication of the degree of potential malignancy in breast cancer can be obtained by means of a histological grading system (Greenough, 1925 ; Patey and Scarff, 1928 ; Haagensen, 1933), and the importance of taking this into account when considering clinical aspects of the disease and results of treatment has been shown elsewhere (Bloom, 1950*a*, 1950*b* and 1956).

The chief aim of this paper is to outline the technique of histological grading of breast cancer that we have used, and to consider its difficulties and limitations. The results of correlating the grade of malignancy with the 5, 10- and 15-year survival rates in a series of some 1400 cases will be presented, and the practical value of grading will be discussed.

### MATERIAL

A series of 1544 female patients with breast cancer seen at the Middlesex Hospital between the years 1936 and 1949 inclusive, and followed for at least five years has been used for this study. They are all cases in which histological material has been available. Some of them have formed the basis for earlier reports on the subject of grading (Bloom, 1950 and 1956).

The majority of the patients (84 per cent) were treated by a radical or modified radical mastectomy with or without ancillary irradiation. Many, especially during the war years (1939–45), were operated upon elsewhere, and were referred to the Meyerstein Institute of the hospital for post-operative radiotherapy.

It is important to note that our cases have been, to some extent, selected since it has been necessary to have histological sections for grading, and therefore the most advanced cases, treated solely by radiotherapy, have not been included except for a few in whom biopsies were taken.

From the total series of 1544 cases we have excluded 135 for the following reasons :

| | Number of cases |
|---|---|
| Total . . . . . . . . . . . . . . . . | 1544 |
| Excluded : | |
| Post-operative deaths . . . . . . . . . . . | 17 |
| Air-raid casualties (1940–45) . . . . . . . . . | 4 |
| Untraced at 5 years . . . . . . . . . . . . | 15 |
| No evidence of frank carcinoma in sections available (including intra-duct papilloma, proliferative mastitis and adenoma of the nipple) | 49 |
| Bilateral carcinoma of the breast (simultaneous and successive) . . | 26 |
| Impossible to grade . . . . . . . . . . . . | 19 |
| Sarcoma . . . . . . . . . . . . . | 4 |
| Squamous cell carcinoma . . . . . . . . . . . | 1 |
| Total . . . . . . . . . | 135 |
| Remaining for consideration . . . . . . . . . . | 1409 |

Bilateral carcinoma of the breast was considered to be a special problem and was excluded from the general series. We failed to grade 19 cases because the only sections of tissue available were either too small, too degenerate, or were prepared by the frozen section technique. The difficulties met with in grading will be dealt with later.

It is a tribute to the Follow-up Department of the hospital that, in spite of the war years occurring in the period covered by this study, only 15 patients could not be traced at 5 years (i.e. 1 per cent of the total series). Patients treated between 1936–45 were available for 10-year results, and of 823 cases only 9 were lost in the 5- to 10-year interval. Fifteen-year results are based on 362 patients treated between 1936–40, and of these 3 remain untraced at 15 years.

### *Method of Grading*

The epithelial elements of the tumour are used for grading according to the method of Patey and Scharff (1928), which is based on the principles formulated by Greenough (1925). The three histological factors studied have been presented elsewhere (Bloom, 1950*a*) and are briefly as follows :

(*a*) Degree of structural differentiation as shown by the presence of tubular arrangement of the cells.

(*b*) Variation in size, shape and staining of the nuclei.

(*c*) Frequency of hyperchromatic and mitotic figures.

Having assessed each of these factors separately the potential malignancy of the tumour is determined from the composite histological picture. The tumour is placed in one of three grades of malignancy, namely, low (Grade I), intermediate

(Grade II) or high (Grade III) as in the classification used by Greenough (1925), Patey and Scarff (1928) and Haagensen (1933). These categories give a good correlation with clinical outcome (Bloom, 1950*a* and 1956) and have the advantage of simplicity over more elaborate methods of grading.

We have found a simple numerical system useful in deciding into which grade a particular tumour should be placed. Points are awarded according to whether each of the three histological factors (tubule formation, pleomorphism and mitoses) is present in slight, moderate or marked degree.

*Differentiation or tubule formation.*—A high degree of differentiation, which is considered to indicate a favourable prognosis, is shown by well-marked tubular or acinar arrangement with cells grouped more or less regularly around a central space. This feature is usually best seen in the more central portions of the growth. Clefts in the tissue, probably caused by shrinkage during processing, can be distinguished from tubules ; in the latter a rim of cytoplasm can usually be made out separating the nuclei from the lumen.

If the greater part of the section shows well-marked tubule formation we award this factor one point. If there is only a moderate attempt at tubule formation two points are given. Sections showing a slight or no attempt at differentiation, the cells growing in sheets or strands, are assigned three points.

*Pleomorphism.*—Since the cell outline is usually indistinct in the histological preparations of most malignant tumours pleomorphism has been judged from the nuclei rather than from the cells as a whole. No attempt has been made to measure nuclear diameters, the assessment of variability being purely subjective.

The greater the nuclear irregularity the worse the prognosis. One point is awarded if the nuclei are fairly uniform in size, shape and staining. If this variation is moderate in degree two points are given. A marked degree of pleomorphism merits three points.

*Hyperchromatic and mitotic nuclei.*—The greater the number present the worse the prognosis. This factor has also been divided into three degrees and given one, two or three points respectively. A " slight degree " implies the presence of only an occasional hyperchromatic or mitotic figure per high-power field. About two or three such figures in most fields examined is considered a " moderate number ". and more than this a " marked number ". Allowance is made for the degree of cellularity of each microscope field since the number of cancer cells in, for example, a " scirrhus " growth with abundant interstitial tissue is less than in a " medullary" type with masses of cells and little intervening stroma, and yet the number of mitoses per cent of cells may be identical. Both mitotic activity and pleomorphism are best assessed from the periphery of the tumour where invasion is taking place.

To obtain a composite picture of a particular section the points allocated to each of the three histological factors are added together, making a possible total of 3 to 9, the smallest number representing the lowest degree of malignancy. We have followed Scarff in cutting this malignant scale into approximately three equal lengths, the divisions being placed between 5 and 6, and between 7 and 8 thus :

Points

| 3 4 5 | 6 7 | 8 9 |
|---|---|---|
| Low (Grade I) | Intermediate (Grade II) | High (Grade III) |

Tumours with 3, 4 or 5 points are classified as being of low malignancy or Grade I, those with 6 or 7 points of intermediate malignancy or Grade II, and those with 8 or 9 points of high malignancy or Grade III (Figs. 1–16).

The 5-year survival rate for cases according to the total number of points awarded is shown in Table I. It is evident that there is a marked difference in prognosis between cases on either side of the scale divisions. The prognosis is uniform within each of the Grade II and Grade III groups. Some difference exists in Grade I ; 85 per cent of patients with a total of only 3 points survived compared with 72 per cent of those with 5 points.

TABLE I.—*Survival Rates According to the Histological Scale of Malignancy*

| | Total points | | | | | | |
|---|---|---|---|---|---|---|---|
| | 3 | 4 | 5 | 6 | 7 | 8 | 9 |
| Grade . . . | | I (Low) | | II (Intermediate) | | III (High) | |
| Number of cases . . | 39 | 121 | 202 | 307 | 333 | 335 | 72 |
| 5-year survivors . . | 33 | 93 | 146 | 141 | 157 | 106 | 24 |
| ,, survivors % . | 85 | 77 | 72 | 46 | 47 | 32 | 33 |

The three classes of tumour are not disparate pathological entities, the lines of cleavage between the grades merely indicating arbitrary divisions of what is, in fact, a continuous scale of malignancy. Whilst neoplasms at either end of the scale are easily recognised, some on the border lines may be more difficult to classify, and not infrequently it is a matter of opinion into which grade they should be placed.

We have described a numerical point system here in the belief that it may be of value to those who wish to grade breast cancer. This does not mean that we ascribe mathematical accuracy to grading. The points system is merely an aid, and we have found it useful in demonstrating the technique of grading to others. With experience it is possible to classify most tumours directly without first having to award points for each factor.

Although a definite histological grading system is not widely used for breast cancer in this country, it is frequently stated that tumours exhibiting well-marked tubule formation, and often referred to as " adenocarcinoma ", are of low malignancy and carry a good prognosis. With the present system of grading, however, it is possible to place such a tumour not in Grade I, but in the intermediate Grade II if there is considerable nuclear irregularity and if frequent mitoses are present (Fig. 11 and 12). Conversely, a growth showing no attempt at tubule formation is frequently reported as an " undifferentiated spheroidal cell carcinoma" and is usually considered to carry a bad prognosis. However such a tumour may be placed in Grade I if the nuclei are uniform and mitoses and hyperchromatic nuclei are rare (Fig. 5 and 6).

The incidence of the three grades of tumour in this series is shown in Table II. A little less than half the cases (45 per cent) were placed in the intermediate grade of malignancy, the remaining cases being approximately equally divided between Grades I (26 per cent) and III (29 per cent).

TABLE II.—*Incidence of Histological Grades*

| Grade | Cases | % |
|---|---|---|
| I . . | 362 | 26 |
| II . . | 640 | 45 |
| III . . | 407 | 29 |
| Total . . | 1409 | 100 |

### *Difficulties Encountered in Grading*

### 1. *Histological preparations*

We have mostly used paraffin-embedded sections stained with haemotoxylin and eosin. It may be possible to grade tumours on good frozen sections, but generally speaking they are too thick and the nuclear detail is seldom definite. Poor technique in preparing sections leads to difficulties in grading. In 13 cases in this series we could not estimate the degree of malignancy because the cells appeared too degenerate, and in some instances even the structural pattern was obscured, presumably due to inadequate fixation.

### 2. *Variation in histological appearance in different parts of the primary tumour*

This is the argument generally advanced by the opponents of grading of breast cancer, although they may accept grading of tumours in other sites such as the rectum, bladder and skin. It is possible that many of the difficulties in carcinoma of the breast have arisen from trying to grade solely by tumour pattern without taking into account the actual cytological features. Tumours can readily be found in which, for example, one microscopic field contains intraduct tumour, another field shows invasive carcinoma with attempted tubule formation, while yet a third is occupied by masses or columns of invading cells. In most cases however, although variation may be present in the histological picture a definite total individual pattern can be recognised. Furthermore, in spite of different forms of cellular arrangement the degree of pleomorphism and the frequency of hyperchromatic and mitotic figures usually show little variation in any one tumour.

Although some idea of the malignancy of a breast tumour can often be obtained from small biopsy specimens, grading should be carried out on sections of reasonable size. Generally speaking a piece of tissue about 1·5–2 cm. square will show the full range of tumour pattern. In most tumours one section is usually sufficient for grading ; sections cut from different parts of the same tumour have shown a comparable grade of malignancy (Bloom, 1950*a*). However, in the case of large growths it is possible that two or perhaps three sections may be required before one can be satisfied that the full histological picture has been seen.

A few cases have shown a definite variation in grade as opposed to mere variation in structural pattern even in the same histological section (Fig. 17–19). In these we have given most weight to the most malignant part of the growth. This problem was studied by Haagensen (1933) who examined an average of 8 sections from each tumour. In only 11 per cent of his 164 cases did he find differences in structure great enough to create difficulties in grading, and he overcame this by classifying the tumour on the basis of its predominant features.

3. *Variation in grade between primary tumour and metastases*

If the histological grade of malignancy in metastases from carcinoma of the breast varies from that of the primary, then prognosis based upon the appearances of the latter may prove misleading. This would apply especially to Grade I tumours if their metastases underwent a change to the more malignant Grade II or even Grade III type. In such cases the possibility of a favourable prognosis suggested by the histological appearance of the primary growth may well have to be modified because of the higher grade in the metastases.

We have studied this question of a change of grade in 397 unselected cases treated between 1943 and 1949, in which the axillary lymph nodes were invaded and the histological sections were available for examination. In 82 per cent of cases the grade of the metastases was identical with that of the primary tumour, of a higher grade in 12 per cent, and of a lower grade in 6 per cent (Table III). Similar results were obtained by Haagensen (1933) who, in 103 cases, found the same grade in the glands as in the breast tumour in 71 per cent, a higher grade in 19 per cent, and a lower grade in 10 per cent. Patey and Scarff (1929) in a series of 110 cases found only one instance of a greater degree of malignancy in axillary metastases. Constancy of grade in glandular metastases is illustrated in Fig. 20–22, and an increase of malignancy in Fig. 23 and 24.

TABLE III.—*Comparison of Histological Grade of Malignancy in Axillary Metastases with the Primary Breast Tumour*

| Grade of metastases in axillary nodes | Number of cases | % |
|---|---|---|
| Identical with primary tumour | 325 | 81·9 |
| Higher than primary tumour | 49 | 12·3 |
| Lower than primary tumour | 23 | 5·8 |
| Total | 397 | 100·0 |

The grade of distant metastases may show remarkably little change, even when there has been an interval of many years between the removal of the primary tumour and the appearance of the secondary deposits (Fig. 25 and 26). An alteration of histological maligancy, however, has been noted in some cases that we have studied, and this change may be influenced by the actual site of the metastasis. Willis (1932) found that secondary deposits in the liver show greater mitotic activity than the primary growth, presumably because of the abundant nutrient material available in this organ. An alteration in the grade of metastases in the liver, however, would not affect the prognosis materially because patients with hepatic involvement seldom live for more than a few months. On the other hand, the presence of metastases in bone and in certain viscera such as the lungs or brain of patients with breast cancer is occasionally compatible with a survival of many months or even a number of years, presumably if the tumour remains of low grade malignancy ; in such cases an increase in malignancy may shorten life to but a few months.

Of greater significance in prognosis are possible changes in malignancy of residual tumour in the breast following radiotherapy, or in the chest wall after surgery, since patients with local recurrences may survive for many years after treatment. The histological picture of local recurrences is in our experience similar to that of the original tumour in most cases (Fig. 27 and 28).

To sum up : although a change in histological grade of malignancy may occur in breast cancer, the majority of cases appear to remain remarkably constant in grade. In some of our cases where grading and the clinical course following treatment have been at variance, the reason may lie in an alteration of maligancy in residual tumour, local recurrences, regional lymph node deposits or distant metastases.

4. *Tumour degeneration not due to faulty fixation*

(a) *Spontaneous degeneration.*—Rapidly growing tumours often show areas of necrosis and this may lead to difficulties in grading. In such cases, however, it is generally possible to find areas of viable-looking tissue suitable for grading in blocks cut from the growing edges of the tumour.

Occasionally, many large, irregular, hyperchromatic nuclei are seen in breast tumour sections and may represent a degeneration phenomenon. Nevertheless, this feature appears to be associated with tumours of high malignancy, and we have taken it into account when considering the degree of pleomorphism and frequency of hyperchromatic nuclei. (Fig. 29).

(b) *Degeneration following radiotherapy* was studied in 60 cases of the 1943–49 period who had completed a course of pre-operative deep x-rays, usually one to three months before operation. This rarely produced such a marked degree of irradiation change that grading became impossible. In most instances there were residual areas of viable-looking tumour cells which could be graded, although sometimes with difficulty. When the primary tumour was totally unsuitable for grading (7 cases), and the axillary glands were invaded we have graded the case on the latter which often appeared to be less affected by irradiation.

5. *Intra-duct carcinoma and " comedo-carcinoma "*

Sections from 8 cases in this series showed such a marked degree of atypical epithelial proliferation within intact ducts as to warrant the term " intra-duct carcinoma ". We have tried to grade these cases, but have not included them in the general results. Obviously if the tumour is confined to the ducts at the time of the mastectomy the prognosis will be good, but it is difficult to be certain of this unless serial blocks have been cut throughout the tumour area. Cases of intra-duct carcinoma showing undoubted areas of stromal invasion were included as frank carcinomas in the general series.

" Comedo-carcinoma " is a term little used in this country, but favoured by a number of American authors. Stewart (1950) points out that the term simply denotes a form of non-infiltrating carcinoma within distended ducts, the core of the proliferating cells having undergone a central necrosis and being expressible as a yellow paste. Haagensen (1933) placed tumours with a " comedo " pattern in his group of low malignancy (Grade I) cases, irrespective of their cytological features, but only 4 of his 10 cases in this group survived 5 years following radical mastectomy. We have not distinguished between " comedo-carcinoma " and intra-duct carcinoma, and of our 8 cases none were classified as Grade I, 5 as Grade II, and 3 as Grade III. Six cases were alive at 5 years.

6. *Colloid carcinoma*

The histological picture of islands of tumour cells in a sea of mucinous material was seen in 22 of our cases. Difficulties in grading occurred in some instances when only small numbers of cells were present, not infrequently with degenerative changes. Tumours with scanty colloid were not considered separately from the main group of cases. Haagensen (1933) and also Gricouroff (1948) arbitrarily placed the colloid type of carcinoma in the lowest grade of malignancy (Grade I).

These cases were graded no differently from the others, but we noted the fact that colloid material was abundant. Most of the tumours showed attempts at tubule formation with moderately regular nuclei and relatively few mitoses, and were therefore placed in Grade I. A few cases were classed as Grade II ; none were Grade III. The prognosis for this group of tumours is shown in Table IV.

TABLE IV.—*Prognosis for Colloid Carcinoma*

| | 5-year results | | 10-year results | |
|---|---|---|---|---|
| Grade | Cases | Survivors | Cases | Survivors |
| I | 15 | 11 (73%) | 7 | 4 |
| II | 7 | 5 (71%) | 2 | 2 |
| III | 0 | 0 | 0 | 0 |
| Total | 22 | 16 (73%) | 9 | 6 (67%) |

RESULTS

In one-third of the cases the tumours were graded by each observer independently of the other and the results were later cross-checked : agreement was found in over 90 per cent of cases. Where a difference of opinion persisted after discussion we usually noted considerable degeneration in the sections, probably due to either faulty fixation or pre-operative irradiation. When degeneration was marked throughout the section the case was excluded. A further third of the cases was graded by W.R., and the remaining third (which formed the basis of an earlier report (Bloom, 1950)) by H.B. Only when grading had been completed were the patients' notes consulted for clinical details.

It is not our purpose here to discuss the treatment of breast cancer. The aim is to draw attention to the relationship between the histological appearance of the tumour and prognosis. The statistics which follow are not the general results obtained by the Middlesex Hospital in the treatment of this disease, because there has been, as already stated, selection of cases by the necessity for having histological material for grading.

Treatment of the patients in this series was not uniform, nor were all the cases first seen at the Middlesex Hospital, many patients being referred from other hospitals for post-operative radiotherapy. Of the total number 442 were treated by surgery alone, 936 by surgery and pre- or post-operative radiotherapy, and 31 by irradiation alone. A radical or modified radical mastectomy was performed in the majority (84 per cent) of cases. This variation in treatment, however, does not appear to materially influence the general conclusions reached from relating histological grade and prognosis (Bloom, 1950*a*).

Prognosis has been gauged by the 5-, 10- and 15-year survival rates. The term " survival rate " as used here does not necessarily imply freedom from cancer ; it merely indicates the percentage of patients actually alive. The 5-year results according to grade for all treatments are shown in Table V. A good correlation between histology and survival has been obtained, there being more than twice the number of survivors with Grade I tumours than with Grade III tumours.

The 10-year results for patients treated between 1936 and 1945 are also presented in Table V, and show almost three times as many survivors in Grade I as in Grade III. This table, in addition, contains the 15-year results for 359 patients treated during 1936 and 1940. Here the percentage of survivors with carcinomas of low malignancy is three times that of patients with Grade III tumours. The survival rate of Grade III cases at 5 years is comparable with that of Grade I cases at 15 years.

TABLE V.—*Grade and Prognosis* (5-, 10- *and* 15-*year results*)

| Grade | 5-year results: Number of cases (1936–49) | Survivors | % | 10-year results: Number of cases (1936–45) | Survivors | % | 15-year results: Number of cases (1936–40) | Survivors | % |
|---|---|---|---|---|---|---|---|---|---|
| I | 362 | 272 | 75 | 215 | 113 | 53 | 96 | 30 | 31 |
| II | 640 | 298 | 47 | 349 | 94 | 27 | 162 | 29 | 18 |
| III | 407 | 130 | 32 | 250 | 47 | 19 | 101 | 10 | 10 |
| Total | 1409 | 700 | 50 | 814 | 254 | 31 | 359 | 69 | 19 |

The results for the three types of tumour are shown graphically in Fig. 30. During the first five years the number of survivors with Grade II or Grade III carcinomas falls steeply. After this time the decline becomes less rapid and approaches that for the expected survival rate for women of a similar age distribution. Grade II and Grade II cases surviving longer than 5 years may be comprised of (*a*) clinical cures, (*b*) patients in whom there had been either an error in grading

FIG. 30.—Grade and prognosis. Five-, ten- and fifteen-year survival rates (from Table V).

*Note.*—The expected survival curve is based on the Registrar General's English Life Tables, 1951 (H.M. Stationery Office, 1957) for a group of females exposed to all causes of death of the same age distribution as the Grade I cases. The age distribution in each of the three grades of malignancy is comparable.

or alternatively in whom a decrease in malignancy had taken place in metastases, and finally (c) patients with a high degree of natural resistance.

The survival rate for Grade I cases, so promising at 5 years, falls steadily and relentlessly over the entire 15 years. This must indicate a high incidence of distant metastases when the patient is first seen, and it is particularly in this group that deposits may remain latent for a number of years before becoming active. The survival rate for Grade I patients may be compared with the expected survival rate for a group of women of the same age distribution and exposed to all causes of death (Fig. 30).

These results suggest that a 5-year follow-up is more significant in determining the success of treatment for patients with tumours of high malignancy than is an interval of 10 and possibly even 15 years for patients with Grade I tumours.

*Grade, gland involvement and prognosis*

The morbid histologist can give a more accurate indication of possible outcome in breast cancer by considering the histological grade of the tumour together with its extent as determined by the presence or absence of metastases in the axillary lymph nodes. These glands were available for microscopic study in 1143 cases, and invasion was found in 705 (62 per cent), which agrees with the results of other large series (Harrington, 1952 ; Haagensen, 1956).

The 5-, 10- and 15-year survival rates according to grade and axillary metastases are shown in Table VI, and graphically in Fig. 31. There is a pronounced difference

Fig. 31.—Gland involvement, grade and prognosis. Five-, ten- and fifteen-year survival rates (from Table VI).

———— Axillary glands free.
— — — — Axillary glands invaded.

between the number of patients alive with Grade I tumours and lymph nodes which are free from metastases, and those with Grade III tumours and invaded nodes. The difference between these extremes is maintained throughout the prolonged follow-up ; 15 years after treatment half of the former group are still alive compared with only 7 per cent of the latter. It is interesting to find that the prognosis at 5 and also 10 years for Grade I tumours in the presence of axillary metastases is just as good or even better than that for patients without axillary involvement that belong to Grade II or Grade III. The outlook for Grade I cases with positive lymph nodes, however, deteriorates rapidly with the prolonged follow-up, the survival rate falling from 51 per cent at 10 years to 15 per cent at 15 years.

In the absence of axillary metastases there is little difference in prognosis between Grade II and Grade III cases, and this was also the finding in a previous report (Bloom, 1950*a*).

Haagensen (1933) has also used a sytem of grading based on the principles formulated by Greenough (1925). Recently he (Haagensen, 1956) has classified a series of 1003 cases according to grade and axillary involvement, the grading being carried out by Stout at the Presbyterian Hospital. The results, which are shown in Tables VII and VIII, agree very closely with those obtained by Greenough himself (Simmons *et al.*, 1933) and also by Bloom (1950*a*), and with those in the present investigation (Tables V and VI).

TABLE VI.—*Gland Involvement, Grade and Prognosis* (5-, 10- *and* 15-*year Results*)

| | | 5-year results | | | 10-year results | | | 15-year results | | |
|---|---|---|---|---|---|---|---|---|---|---|
| | | | Survivors | | | Survivors | | | Survivors | |
| Glands | Grade | Cases | | % | Cases | | % | Cases | | % |
| Not invaded | I | 147 | 126 | 86 | 94 | 57 | 61 | 43 | 21 | 49 |
| | II | 205 | 139 | 68 | 110 | 52 | 47 | 63 | 18 | 29 |
| | III | 86 | 55 | 64 | 57 | 24 | 42 | 24 | 6 | 25 |
| | Total | 438 | 320 | 73 | 261 | 133 | 51 | 130 | 45 | 35 |
| Invaded | I | 145 | 96 | 66 | 81 | 41 | 51 | 34 | 5 | 15 |
| | II | 324 | 108 | 33 | 178 | 25 | 14 | 79 | 9 | 11 |
| | III | 236 | 44 | 19 | 138 | 12 | 9 | 58 | 4 | 7 |
| | Total | 705 | 248 | 35 | 397 | 78 | 20 | 171 | 18 | 11 |
| Grand Total | | 1143 | 568 | 50 | 658 | 211 | 32 | 301 | 63 | 21 |

TABLE VII.—*Grade and Prognosis* (*Haagensen*, 1956)

| | | 5-year clinical cures | |
|---|---|---|---|
| Grade | Cases | | % |
| I | 84 | 66 | 79 |
| II | 422 | 203 | 48 |
| III | 597 | 196 | 33 |
| Total | 1103 | 465 | 42 |

### DISCUSSION

The degree of malignancy in carcinoma of the breast is reflected in the histological structure of the tumour and this feature forms the basis for a classification of the disease. As a guide to prognosis, however, histological grading in our hands offers no more information than does clinical staging which is in general

TABLE VIII.—*Gland Involvement, Grade and Prognosis* (*Haagensen*, 1956)

| Glands | Grade | Cases | 5-year clinical cures | % |
|---|---|---|---|---|
| Not invaded | I | 52 | 45 | 87 |
| | II | 165 | 114 | 69 |
| | III | 202 | 121 | 60 |
| Invaded | I | 32 | 21 | 66 |
| | II | 257 | 89 | 35 |
| | III | 395 | 75 | 19 |

use at the present time. Why then grade breast cancer ? Earlier work has shown that the classification of carcinoma of the breast, based solely upon a system of staging, produced groups of cases which were not strictly comparable, and this was thought to account for at least some of the variable results of treatment obtained in this disease (Bloom, 1950*a*). An attempt was made at that time to overcome this difficulty by introducing a clinico-pathological classification which embraced both clinical staging and histological grading, and the results obtained showed that this combined approach offered a more accurate guide to prognosis than did either method alone.

Our concept of three grades of malignancy in breast cancer is supported by the results in Table V, there being from two to three times as many survivors with Grade I as with Grade III tumours 5 to 15 years after treatment. Furthermore the value of grading is greatly enhanced by taking into account the state of the axillary lymph nodes (Table VI). We have been fortunate in also being able to study a series of untreated cases of breast cancer observed at the Middlesex Hospital between 1805 and 1920, in collaboration with Dr. E. J. Harries. Among these were 63 patients, seen in the later years, from whom biopsies had been taken, and which we have graded in the same way as the treated series, before referring to the clinical details. Survival rate has been plotted against the duration of life from the *onset of symptoms* for each of the three grades (Fig. 32). These results add further weight to the importance of morbid histology in determining outcome in carcinoma of the breast and also support the accuracy of the grading system we have used.

Grading reflects the potential malignancy of the tumour and is of value for two reasons. First, it provides a measure of the probable extent of a tumour when the patient is first seen, the most malignant growths having spread furthest. Thus, 73 per cent of Grade III tumours compared with 50 per cent of Grade I have axillary metastases at the time of treatment (from Table VI). The difference between these two types of tumour is greater in fact than these figures suggest since patients with Grade III cancer tend to seek advice earlier than those with Grade I lesions ; the average duration of symptoms for the former is 7 months compared with 10 months for the latter (Bloom, 1950*b*). Grading may therefore succeed just where staging fails, by indicating the likelihood of occult metastases being present in regional lymph nodes and distant organs which would not be detected by the ordinary methods of staging.

The second factor of importance in grading is suggested by the persistent steady decline in survival rate up to 15 years for patients with Grade I tumours

FIG. 32.—Untreated breast cancer—grade and prognosis. Sixty-three cases with histology observed at the Middlesex Hospital.

(Fig. 30). This points to a high incidence of distant metastases when the patient is first seen for even tumours of low malignancy, a view which is supported by the fact that 50 per cent of such cases have axillary deposits at the time of treatment. Although we have stated that Grade III carcinomas are more likely to have produced metastases than the essentially more benign Grade I tumours, we consider that the real value of histological grading lies in providing a guide to the speed with which secondary deposits from breast cancer are likely to develop. Thus, metastases are common in Grade I and Grade III cases, but those in the latter group become active sooner, grow more rapidly and produce symptoms and death earlier. Lenz and Freid (1931), in a series of 36 tumours graded according to Greenough (1925) found the mean interval from treatment to onset of skeletal metastases for Grade I cases to be 46 months compared with only 10 months for Grade III cases. The mean survival after the appearance of such metastases in Grade I patients was 19·7 months compared with 10 months for Grade III patients.

The practical value of grading of breast cancer has been questioned on the grounds that the clinician has to assess the management of the patient by a physical examination, and grading is only carried out on a surgical specimen after treatment has been instituted (Smithers *et al*, 1952). If the only value of grading were to enable cases of breast cancer to be grouped more accurately even after treatment has been carried out, then this alone would warrant its wider use. A more accurate grouping of cases will eventually lead to a more accurate assessment of treatment, and may help to clarify the relative merits of the different lines of approach to operable cases. Grading, however, may also prove of value in considering other aspects of breast cancer management.

At the present time little is known about the relative radiosensitivity of the three grades of tumour, and equally dramatic responses have been observed in well-differentiated Grade I tumours and anaplastic Grade III cases (Bloom, 1956). With further knowledge of tumour response to irradiation it may eventually become possible to assess the kinds of treatment most likely to achieve the best results for certain groups of cases depending not only upon the apparent extent of the growth (stage), but also upon its potential malignancy (grade). If this materialises, then it may become important to know the grade of the tumour *before* treatment is given. As grading can be carried out on biopsy specimens of reasonable size the only objection that remains is that of the possible dangers in breast cancer of biopsy itself. The available evidence shows that such a procedure, if followed by treatment within a few days, does not affect the prognosis adversely (Scheel, 1953 ; Pierce *et al.*, 1956 ; Haagensen, 1956).

A further possible application of histological grading of breast cancer in in the hormonal treatment of advanced cases. First of all, knowledge of the grade of tumour will help to evaluate this treatment more accurately since the natural history of the disease differs greatly for Grade I and Grade III cases (Fig. 32). Secondly, only a small number of patients shows a definite objective response to hormones and, generally speaking, it has been impossible to predict which cases are likely to derive benefit from them. Lowenhaupt and Steinbach (1949), however, in a small series graded according to Greenough's (1925) method attempted to correlate the response of remote metastases of breast cancer to testosterone and oestrogen therapy with the grade of malignancy. They concluded that tumours of low malignancy showed the most favourable responses. Emerson *et al.*, (1953), also using Greenough's method of grading in 87 cases, reported that 60 per cent of Grade I cases showed a good or excellent response to hormonal treatment compared with only 22 per cent of Grade III cases. Similarly, MacDonald, Davis and Jacobson (1952) showed that the average longevity in advanced cases following oestrogen or androgen administration was almost four times greater for Grade I than for Grade III patients (19·5 months compared with 5·25 months).

Hochman (1952) has reported the work of the late Professor Halberstaeter of Jerusalem who correlated the effects of an artificial menopause in breast cancer patients with the histology of the primary tumour. Improvement occurred much more frequently in the more differentiated or " adenocarcinoma " group. Hochman suggests that a study of histology may result in more precise indications for inducing an artificial menopause in the management of breast cancer. More recently, Dao and Huggins (1955) express the view that improvement in advanced cases following bilateral adrenalectomy is more likely to be found in patients with tumours showing tubule formation. Of 38 cases with a favourable response to the operation 73 per cent had tumours described as " adenocarcinoma ", and only cases with this type of growth enjoyed profound and prolonged regression. No regression was seen in patients with undifferentiated carcinoma. On the other hand, Cade (1954) reports that all histological types may respond favourably to adrenalectomy and so far this appears to be general experience.

In order to encourage the wider use of histological grading it is essential to maintain simplicity in whatever method is used. Complicated systems of classification are time-consuming, of doubtful value, make the comparison of results difficult, and do much to deter pathologists from attempting to grade tumours. The system of grading employed in this study is simple to apply and has proved an effective guide to prognosis.

It is important to remember that grading is indeed only a guide, and it should not be regarded as a method of predicting outcome with any degree of mathematical accuracy. Some disappointmnts and surprises must be expected, especially when considering individual cases. For example, the patient with the highly malignant-looking Grade III breast cancer in Fig. 33 and 34, which shows no attempt at tubule formation, a gross degree of pleomorphism and numerous hyperchromatic and mitotic nuclei, remains alive and free from recurrence 12 years after radical mastectomy. In contrast to this case Fig. 35 and 36 show a relatively benign-looking Grade I tumour with well-marked tubules, regular nuclei and very few mitoses. This carcinoma which had axillary deposits was operable, but the patient died with skeletal metastases 22 months after radical mastectomy. Striking anomalies like these, however, are rare.

Harrington (1953) has made a special study of the extended follow-up of a large number of breast cancer cases treated at the Mayo Clinic. In his series there were 35 patients who survived 35 years with or without axillary metastases at the time of the radical mastectomy. Fifteen of these cases had been graded according to the method of Broders, and none were classified as being of low malignancy. Eleven of the 15 belonged to Grades 3 and 4. Harrington concluded that some patients with highly malignant tumours and invaded axillary glands may survive for many years following operation. Some of these successes may be accounted for by the occurrence of the so-called " medullary carcinoma with lymphoid infiltrate " which has been shown, in spite of its high degree of potential malignancy, to carry a good prognosis following radical surgery (Moore and Foote, 1949 ; Richardson, 1956).

It is evident that other factors must be concerned in determining the outcome of those patients with breast cancer who do not conform to the expected prognosis based upon the tumours' clinical extent and histological type. It has often been stated that prognosis in this disease is influenced by such features as the age of the patient, pregnancy or lactation, delay in seeking treatment and the site and size of the primary growth. Except for pregnancy and lactation, these factors have not been found, *per se*, to influence prognosis materially (Bloom, 1950*b* ; Haagensen, 1956 ; Kreyberg, 1953 ; Kreyberg and Christiansen, 1953 ; Lewison, 1955). What then are the factors which enable women with advanced highly malignant tumours to survive occasionally for many years? What causes the early death of a number of women with tumours of low grade malignancy which are apparently confined to the breast ?

Stage and grade may tell us a great deal about the tumour itself, but nothing directly about the natural resistance of the patient. MacCarty (1922), in a study of cancer at a number of sites including the breast, concluded that prognosis is determined not only by the degree of cellular anaplasia of the tumour, but also by certain stromal features such as fibrosis, hyalinisation and lymphocytic infiltration which he considered to represent a defensive mechanism by the body. Greenough (1925) and Haagensen (1933) who were able to relate prognosis in carcinoma of the breast to cellular differentiation did not find the stromal features mentioned above of prognostic significance.

More recently, Black, Opler and Speer (1955 and 1956) have graded breast cancer according to the degree of nuclear differentiation and lymphoid infiltration of the primary tumour, and sinus histiocytic reaction of the axillary lymph nodes. The last two factors are considered by Black and his colleagues to represent " tumour retarding " factors of the host. In the presence of well-differentiated

### EXPLANATION OF PLATES

FIG. 1.—Low malignancy (Grade I). Well-marked tubule formation ; nuclei uniform in size, shape and staining ; hyperchromatic and mitotic figures rare. × 45.
FIG. 2.—As for Fig. 1. × 270.
FIG. 3.—Low malignancy (Grade I). A few tubules ; regular muclei ; infrequent hyperchromatic and mitotic figures. × 45.
FIG. 4.—As for Fig. 3. × 250.
FIG. 5.—Low malignancy (Grade I). No attempt at tubule formation ; nuclei uniform ; hyperchromatic and mitotic figures absent. × 45.
FIG. 6.—As for Fig. 5. × 270.
FIG. 7.—Intermediate malignancy (Grade II). Occasional tubules ; moderate nuclear irregularity ; moderate number of hyperchromatic and mitotic figures. × 60.
FIG. 8.—As for Fig. 7. × 250.
FIG. 9.—Intermediate malignancy (Grade II). Moderate degree of tubule formation ; irregular nuclei ; moderate number of hyperchromatic figures and mitoses. × 45.
FIG. 10.—As for Fig. 9. × 270.
FIG. 11.—Intermediate malignancy (Grade II). Well-marked tubule formation ; irregular nuclei ; frequent mitoses. × 45.
FIG. 12.—As for Fig. 11. × 270.
FIG. 13.—High malignancy (Grade III). No attempt at tubule formation ; marked nuclear irregularity ; moderate number of hyperchromatic and mitotic figures. × 45.
FIG. 14.—As for Fig. 13. × 270.
FIG. 15.—High malignancy (Grade III). No tubules ; gross nuclear irregularity ; numerous hyperchromatic and mitotic figures. ×45.
FIG. 16.—As for Fig. 15. × 270.

*Variation of grade in primary tumour*

FIG. 17.—Primary tumour showing area of Grade II carcinoma. No attempt at tubule formation, moderate nuclear irregularity and few mitoses. × 30.
FIG. 18.—Same primary tumour as in Fig. 17. Another area showing Grade I carcinoma with well-marked tubules. × 45.
FIG. 19.—Axillary lymph node. Deposit of Grade II carcinoma identical in appearance with the more malignant part of primary tumour shown in Fig. 17. × 35.

*Constancy of grade in glandular metastases*

FIG. 20.—Primary tumour. Well-differentiated Grade I carcinoma with good tubules, regular nuclei and infrequent mitoses. × 70.
FIG. 21.—Axillary lymph node. Replaced by metastasis of identical grade to the primary tumour (Fig. 20). × 70.
FIG. 22.—Internal mammary lymph node. Small secondary deposit of identical appearance to the axillary metastasis (Fig. 21). × 70.

*Alteration of grade in glandular metastases*

FIG. 23. Primary tumour. Grade I carcinoma showing good tubules, uniform nuclei and infrequent mitoses. × 50.
FIG. 24.—Axillary lymph node. Metastasis of higher malignancy (Grade II) than the primary tumour. Occasional tubules, moderate nuclear regularity and moderate number of hyperchromatic and mitotic figures. × 50.

*Constancy of grade in distant metastases*

FIG. 25.—Primary breast tumour removed by radical mastectomy 1934. Grade I carcinoma with well-marked tubule formation, uniform nuclei and very infrequent mitoses. × 50.
FIG. 26.—Ovarian tumours removed by pan-hysterectomy 1955. Invasion of both ovaries and pelvic peritoneum by adenocarcinoma of similar appearance and grade to the breast tumour removed 21 years previously. × 50.

*Constancy of grade in local recurrences*

FIG. 27.—Primary breast tumour removed by radical mastectomy 1943. Grade I carcinoma with well-marked tubules. × 70.
FIG. 28.—Local recurrence in scar 1947. Grade I carcinoma of identical structure to primary tumour. × 70.

FIG. 29.—Grade III breast cancer showing many large irregular hyperchromatic figures which are usually associated with a high degree of histological malignancy. × 280.

*Anomalies of grading*

FIG. 33.—Breast tumour of high grade malignancy (Grade III) showing no attempt at tubule formation, gross pleomorphism and numerous hyperchromatic and mitotic figures. The patient, however, remains free from recurrence 12 years after radical mastectomy. × 60.
FIG. 34.—As for Fig. 33. × 360.
FIG. 35.—Breast carcinoma of low grade malignancy (Grade I) with well-marked tubule formation, uniform nuclei and infrequent mitoses. The tumour was clinically operable, but the patient died with skeletal metastases 22 months after radical mastectomy. × 80.
FIG. 36.—As for Fig. 35. × 320.

1

2

3

4

5

6

7

8

9

10

11

12

13

14

15

16

17

18

19

20

21

22

23

24

Bloom and Richardson.

25

26

27

28

29

33

34

35

36

nuclei and marked lymphocytic infiltration of the breast cancer together with sinus histiocytosis in the regional nodes, very high survival rates are to be expected without regard to the presence or absence of axillary metastases. The three histological criteria do not bear a fixed relationship to one another so that a tumour of low grade malignancy that kills rapidly may do so because of the patient's poor defensive reaction to the tumour, characterised by the absence of lymphoid infiltration and sinus hyperplasia. At the present time little is known in man about the defensive mechanism to cancer, and whether attempts to combat the invading cells produce changes which can be recognized with ordinary histological techniques. The work of Black, Opler and Speer (1955 and 1956) on " tumour retarding " factors, which has been refuted recently by Berg (1956), awaits confirmation.

We have dealt with the influence of histological grade of malignancy upon the expectation of life of patients with breast cancer. Are there any factors which influence the histological grade of the tumour itself ? Nothing is known at the present time of possible hereditary, racial or geographical factors upon the incidence of tumours of low and high malignancy. We have information, however, on two factors which may affect the type of tumour, namely, age and pregnancy or lactation. Age has been found not to influence prognosis ; just as many tumours of high malignancy as of low malignancy occur in each decade (Bloom, 1950*b*). On the other hand, breast cancer associated with child-bearing carries a poor prognosis (Cheek, 1953 ; White, 1955), and, in a preliminary report on the subject (Bloom, 1955), the majority of tumours were found to be of the highly malignant Grade III type. This is in marked contrast to the present series of general cases where tumours of low and high malignancy have an approximately equal incidence. Perhaps the increased blood supply of the breast or changes in hormonal environment in pregnancy and lactation play a part in determining the high grade of malignancy.

### SUMMARY

Since there is great variation in the behaviour of carcinoma of the breast, even in patients with tumours of comparable clinical extent, we have attempted to recognise different degrees of malignancy in this disease from the histological appearance of the growth.

The tumours of 1409 cases of breast cancer were divided into three grades of malignancy depending upon simple histological criteria and a good correlation with prognosis based upon 5, 10- and 15-year survival rates obtained. The number of survivors with tumours of low grade malignancy was between two and three times greater than those with tumours of high malignancy. A more accurate guide to prognosis can be obtained from histological data by considering the grade of the tumour with the presence or absence of metastases in the axillary lymph nodes. Thus the 5-year survival rate varied from 86 per cent for low grade cases with the axilla free to 19 per cent for high grade cases with the axilla involved. The corresponding figures at 10 years were 61 and 9 per cent, and at 15 years 49 and 7 per cent.

Grading reflects the potential malignancy of the tumour and indicates which cases are more likely to have occult distant metastases at the time of treatment. However, as metastases appear to be common in all three grades of tumour when the patient first seeks advice, the real value of histological grading is in providing a guide to the speed with which such metastases become active, produce symptoms and cause death.

The technique of histological grading has been described in detail and possible difficulties discussed. Although some variation in histological architecture is common in breast cancer a definite individual pattern can usually be recognised in any particular tumour, and in most instances there is little difficulty in deciding into which grade it should be placed. Furthermore, the histological appearance of metastases usually closely resembles that of the parent tumour. In a group of 397 patients the grade of malignancy in axillary lymph node metastases was identical with that of the breast tumour in 82 per cent of cases.

Reference is made to the possible value of grading in determining the most suitable treatment for a particular type of case depending upon the clinical extent of the tumour and its histological type. The use of grading to assist in the evaluation of endocrine therapy is also mentioned.

Brief reference is made to the question of " host resistance " as a possible factor in those patients whose clinical outcome does not conform to the predicted prognosis.

Factors which may influence the histological grade of breast cancer are mentioned. So far, only the rare association of pregnancy and lactation has been found to have any effect, in which the majority of tumours are of high grade malignancy with a correspondingly poor prognosis.

We wish to thank Professor R. W. Scarff for introducing us to his method of histological grading of breast cancer, Professor B. W. Windeyer for encouragement, Dr. P. Strickland for helpful criticism and Dr. J. W. Boag for some statistical advice.

Our gratitude is due to the surgeons of the Middlesex Hospital and War-time Sector Units, and to Professor Windeyer and Miss Margaret Snelling of the Meyerstein Institute of Radiotherapy for allowing us to study cases under their care.

It is a pleasure to acknowledge the assistance given by the pathologists of the many hospitals referring cases for post-operative irradiation, who have allowed us to study the histology of their surgical specimens.

We wish to thank Mr. T. E. Cowan of the Records Department, and Miss J. Chambers of the Follow-up Department of the Hospital for tracing the patients.

### REFERENCES

Berg, J. W.—(1956) *Cancer*, **9**, 935.

Black, M. M., Opler, S. R. and Speer, F. D.—(1955) *Surg. Gynec. Obstet.*, **100**, 543.—(1956) *Amer. J. Clin. Path.*, **26**, 250.

Bloom, H. J. G.—(1950*a*) *Brit. J. Cancer*, **4**, 259.—(1950*b*) *Ibid.* **4**, 347.—(1955) *Ann. Rep. Brit. Emp. Cancer Campgn*, **33**, 30.—(1956) *Brit. J. Radiol.*, **29**, 488.

Cade, Stanford.—(1954) *Ann. R. Coll. Surg. Engl.*, **15**, 71.

Cheek, J. H.—(1953) *Arch. Surg., Chicago*, **66**, 664.

Dao, T. L-Y. and Huggins, C.—(1955) *Ibid.*, **71**, 645.

Emerson, W. J., Kennedy, B. J., Graham, J. N. and Nathanson, I. T.—(1953) *Cancer*, **6**, 641.

Greenough, R. B.—(1925) *J. Cancer Res.*, **9**, 453.

Gricouroff, G.—(1948) *Pr. méd.*, **54**, 638.

HAAGENSEN, C. D.—(1933) *Amer. J. Cancer*, **19**, 285.—(1956) ' Diseases of the Breast '. Philadelphia (Saunders), pp. 133, 522.
HARRINGTON, S. W.—(1952) *J. Amer. med. Ass.*, **148**, 1007.—(1953) *Ann. Surg.*, **137**, 843.
HOCHMAN, A.—(1952) *J. Fac. Radiol. Lond.*, **3**, 199.
KREYBERG, L.—(1953) *Brit. J. Cancer*, **7**, 157.
*Idem* AND CHRISTIANSEN, T.—(1953) *Ibid.*, **7**, 37.
LENZ, M. AND FREID, J. R.—(1931) *Ann. Surg.*, **93**, 278.
LEWISON, E. F.—(1955) *Surgery*, **37**, 479.
LOWENHAUPT, E. AND STEINBACH, H. L.—(1949) *Surg. Gynec. Obstet.*, **88**, 291.
MACCARTY, W. C.—(1922) *Ann. Surg.*, **76**, 9.
MACDONALD, I., DAVIS, F. E. AND JACOBSON, G.—(1952) *Amer. J. Roentgenol.*, **68**, 954.
MOORE, O. S. AND FOOTE, F. W.—(1949) *Cancer*, **2**, 635.
PATEY, D. H. AND SCARFF, R. W.—(1928) *Lancet*, i, 801.—(1929) *Ibid*, ii 492.
PIERCE, E. H., CLAGETT, O. T., MCDONALD, J. R. AND GAGE, R. P.—(1956) *Surg. Gynec. Obstet.*, **103**, 559.
RICHARDSON, W. W.—(1956) *Brit. J. Cancer*, **10**, 415.
SIMMONS, C. C., WRIGHT, J. H., HARTWELL, F. H. AND GREENOUGH, R. B.—(1933) *Amer. J. Cancer*, **19**, 325.
SCHEEL, A.—(1953) *Acta radiol., Stockh.*, **39**, 249.
SMITHERS, D. W., RIGBY-JONES, P., GALTON, D. A. G. AND PAYNE, P. M.—(1952) *Brit. J. Radiol., Suppl.*, No. 4, p. 27.
STEWART, F. W.—(1950) ' Atlas of Tumour Pathology '. Armed Forces Inst. of Pathology, Sect. 9, Fasc. 34, Washington.
WHITE, T. T.—(1955) *Amer. J. Obstet. Gynec.*, **69**, 1277.
WILLIS, R. A.—(1932) *J. Path. Bact.*, **35**, 11.


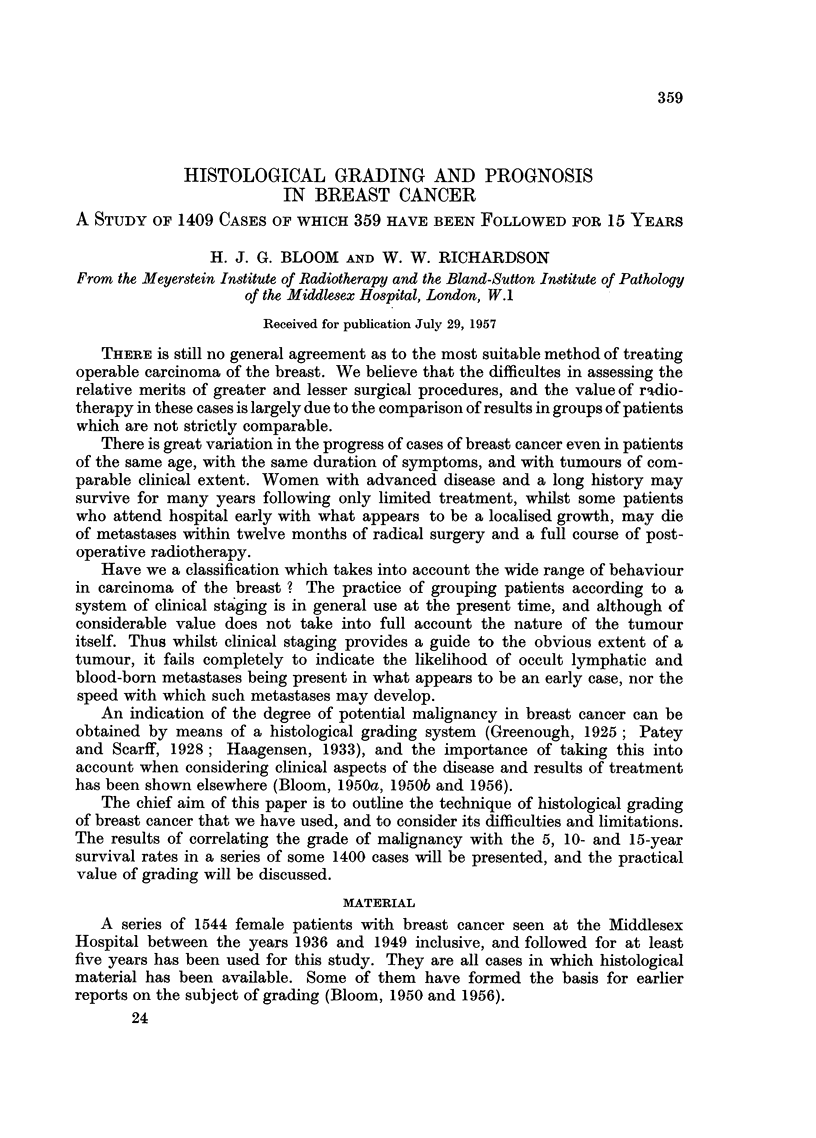

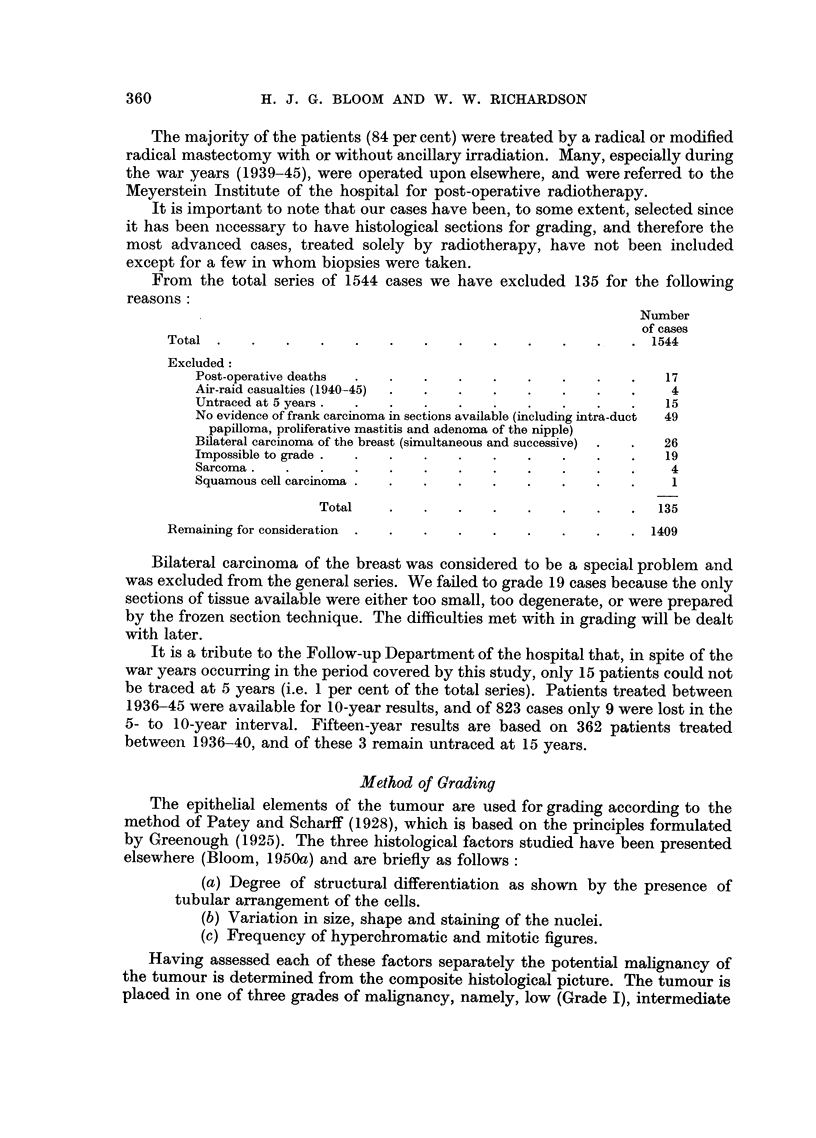

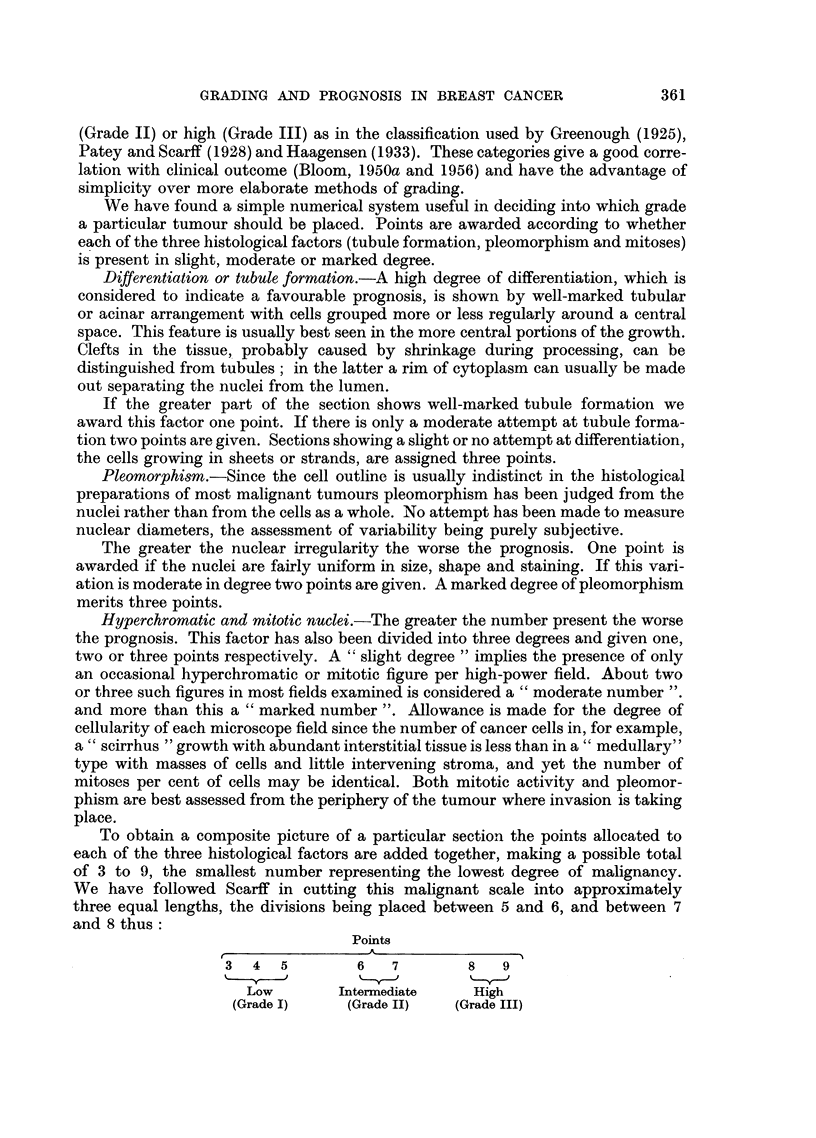

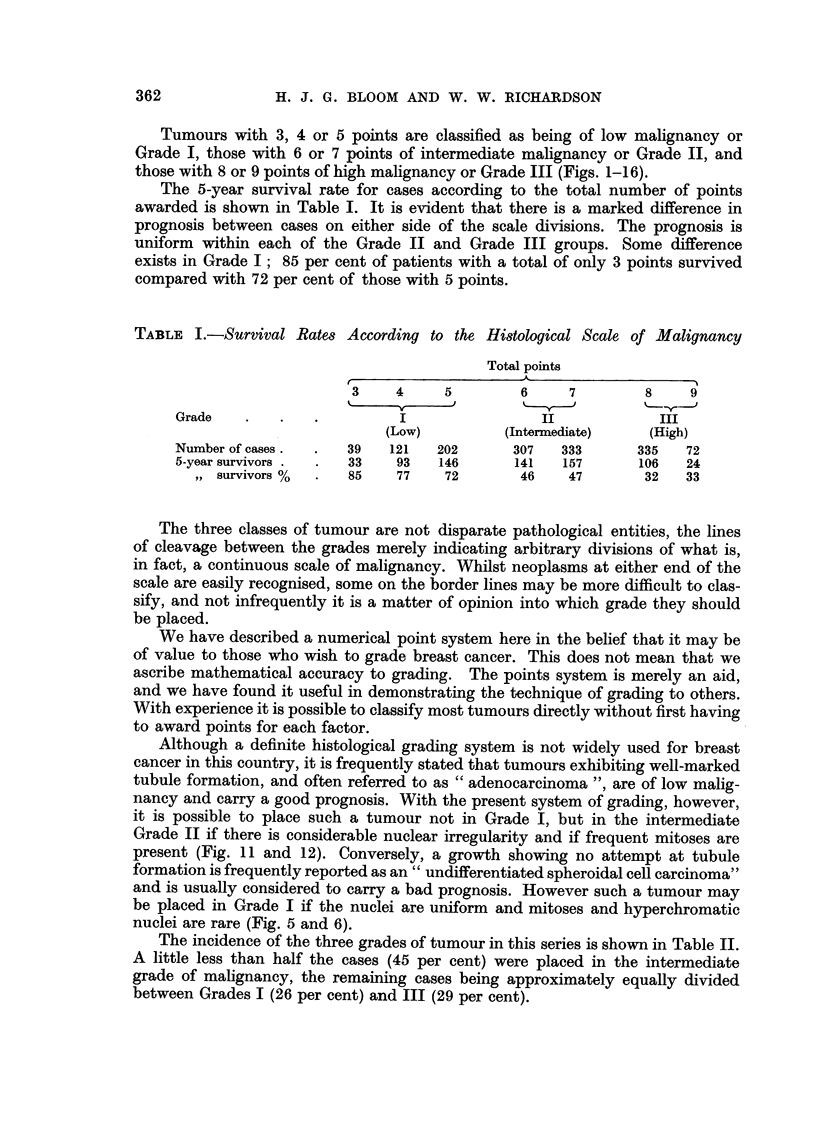

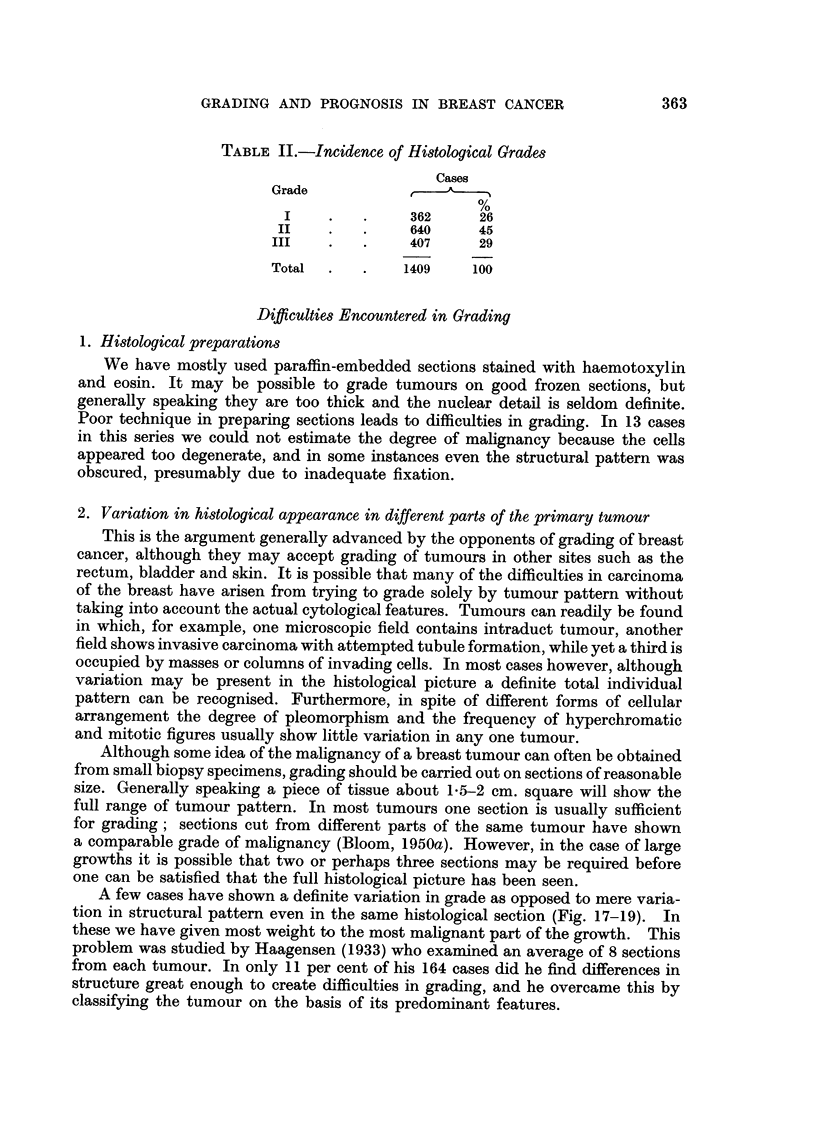

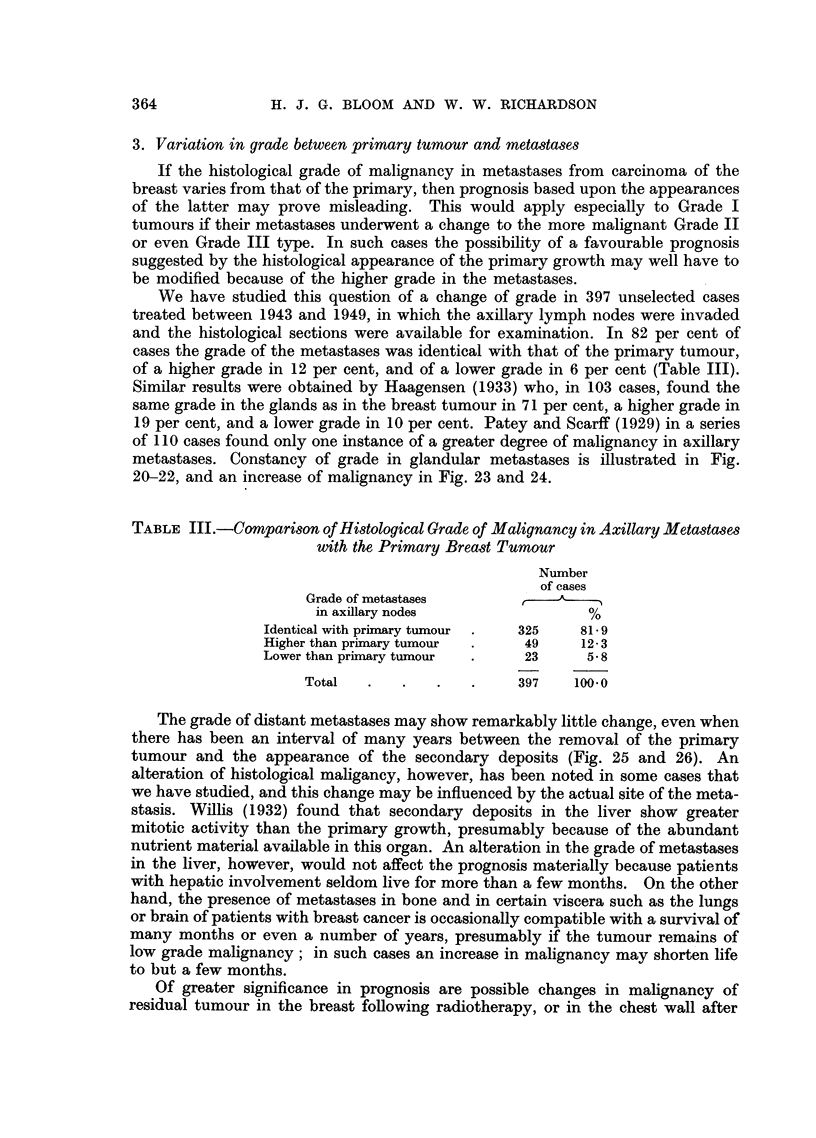

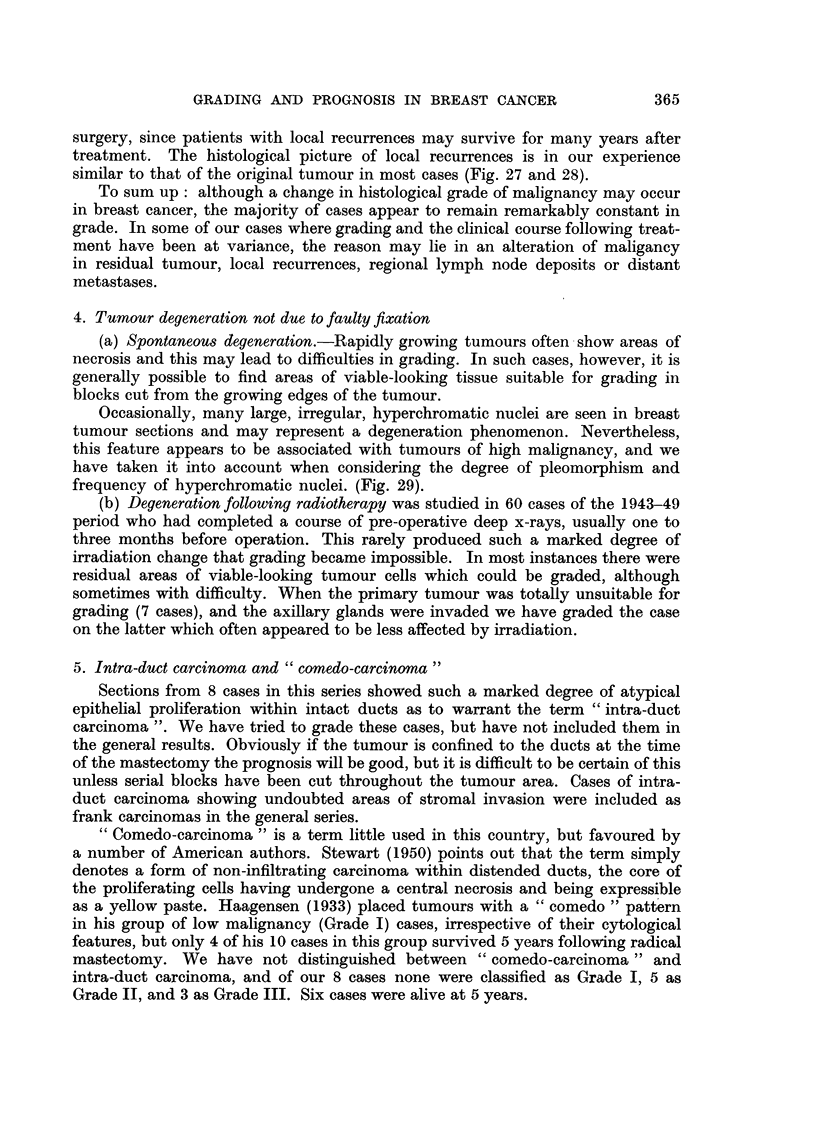

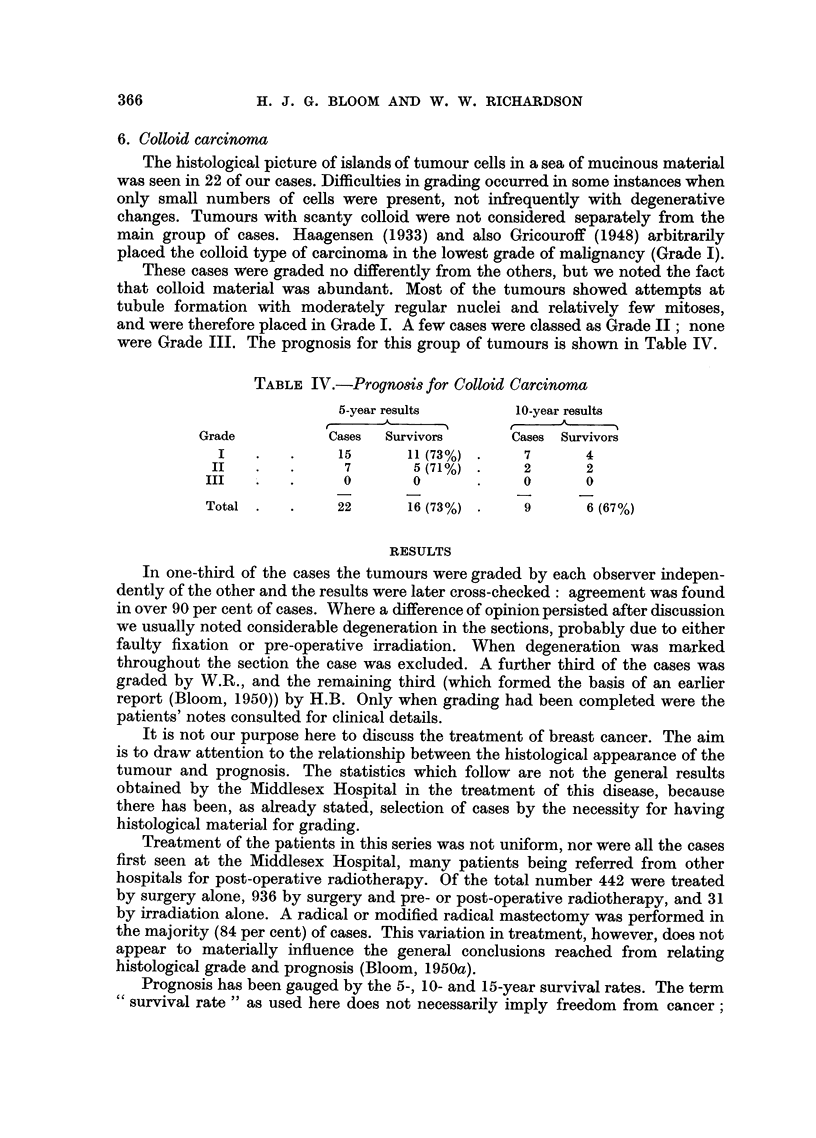

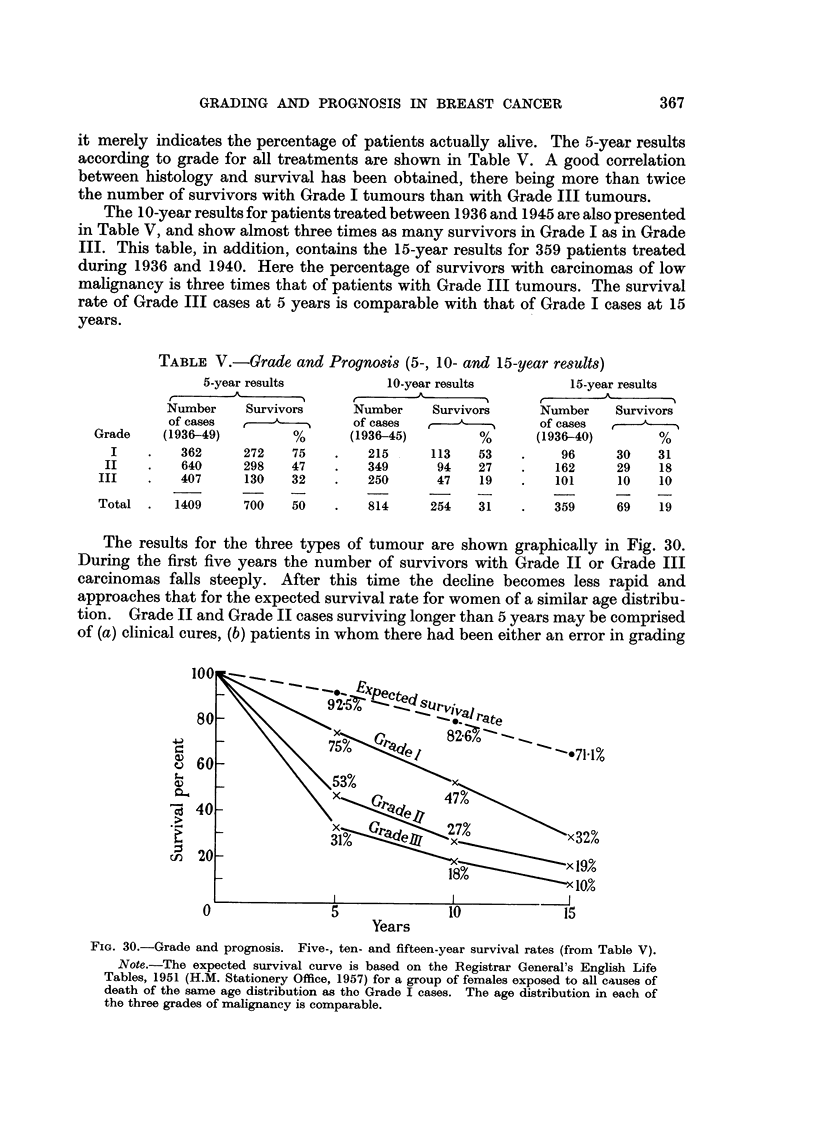

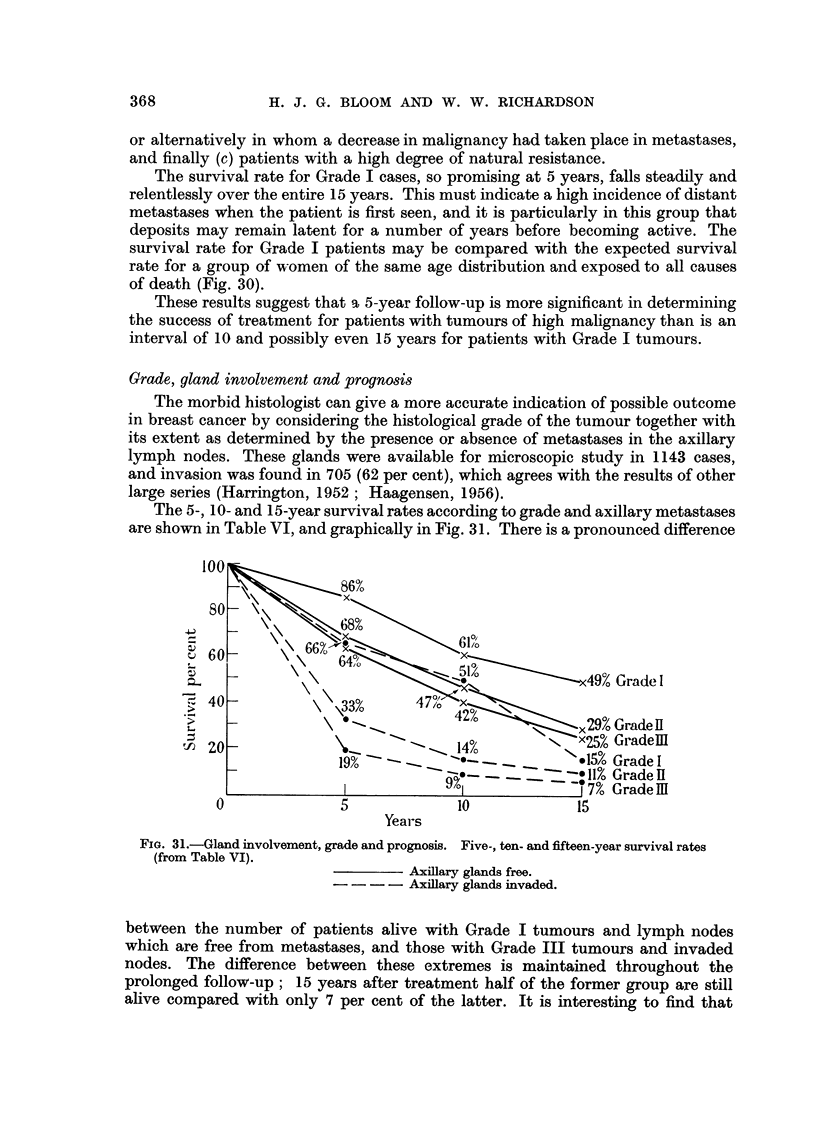

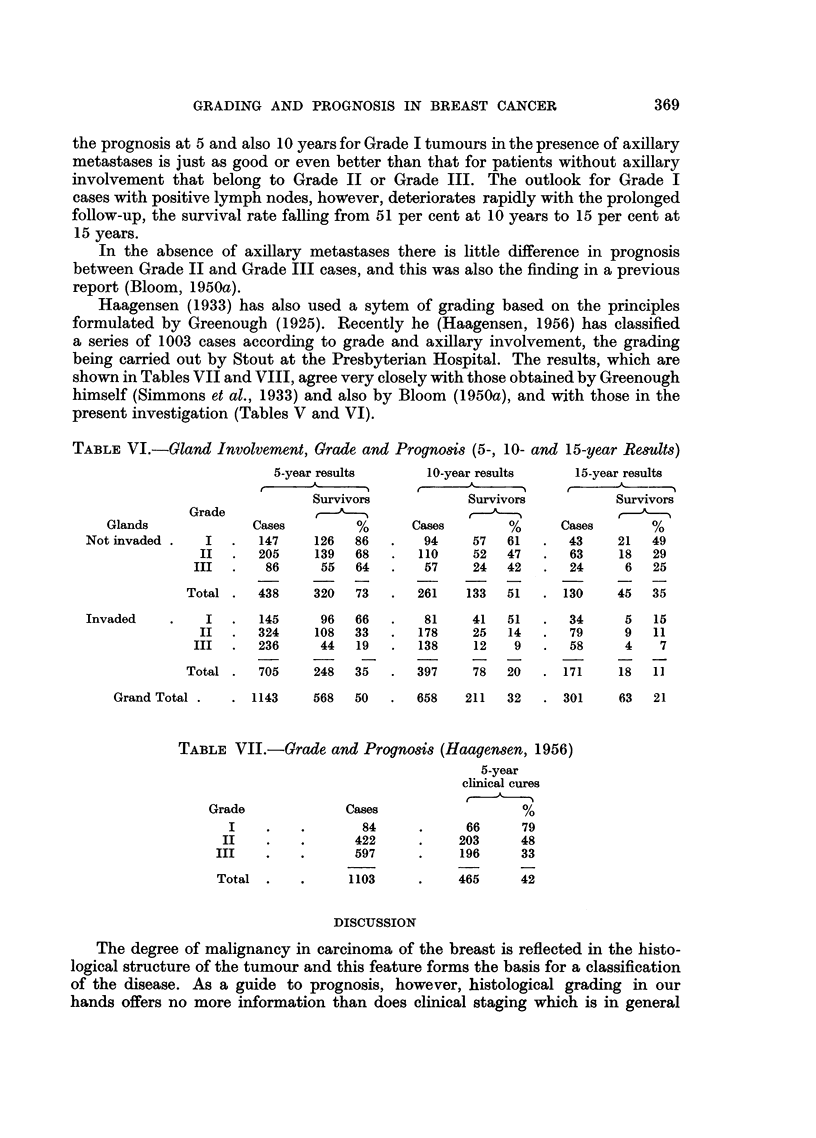

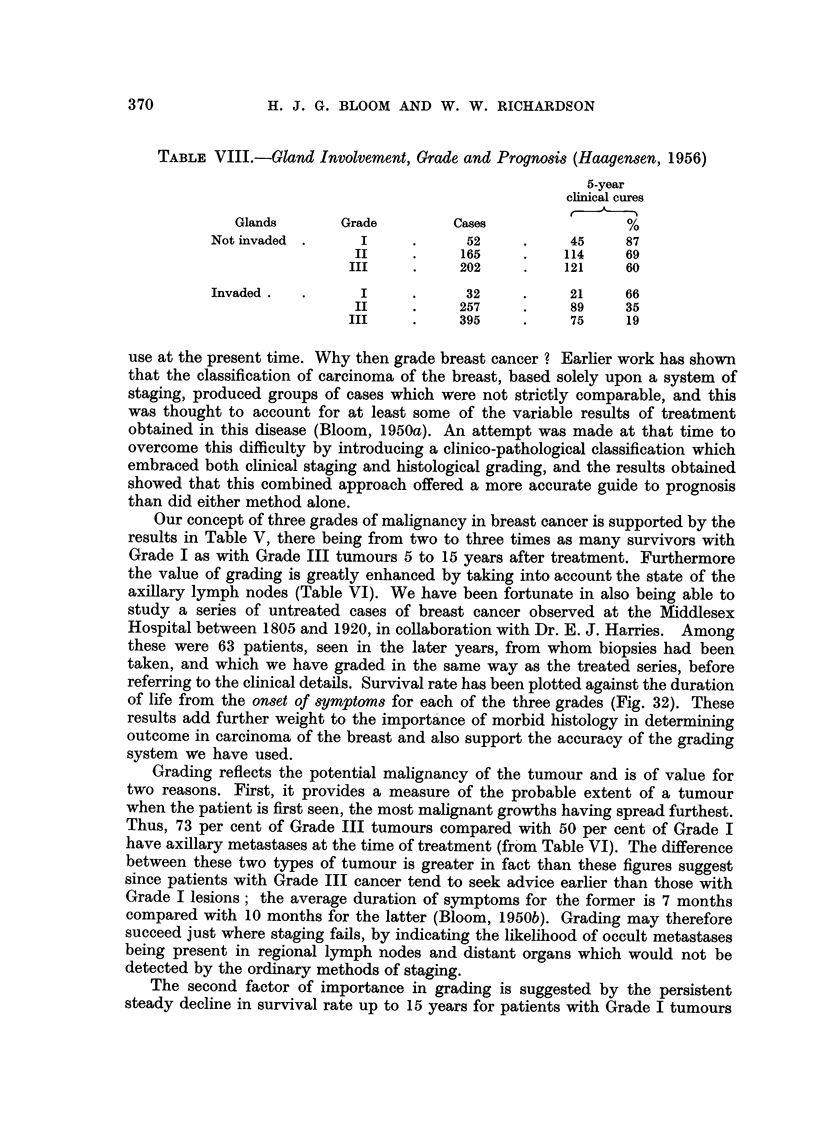

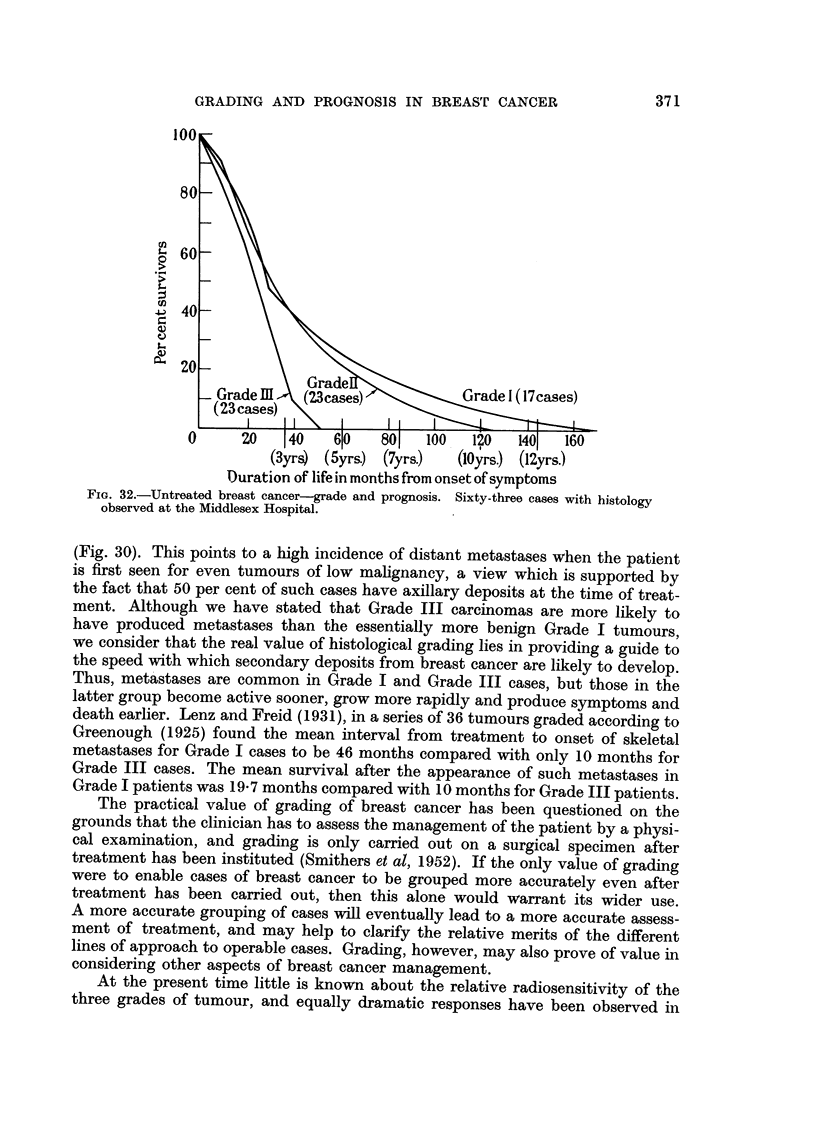

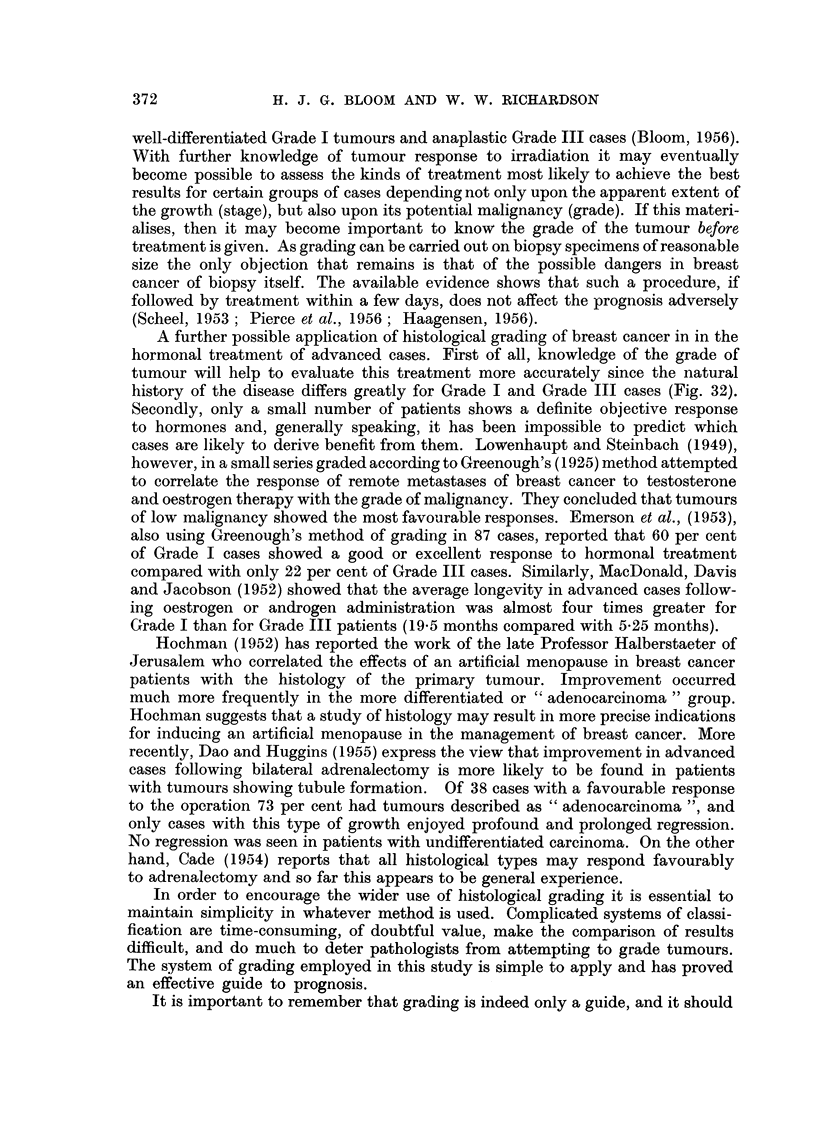

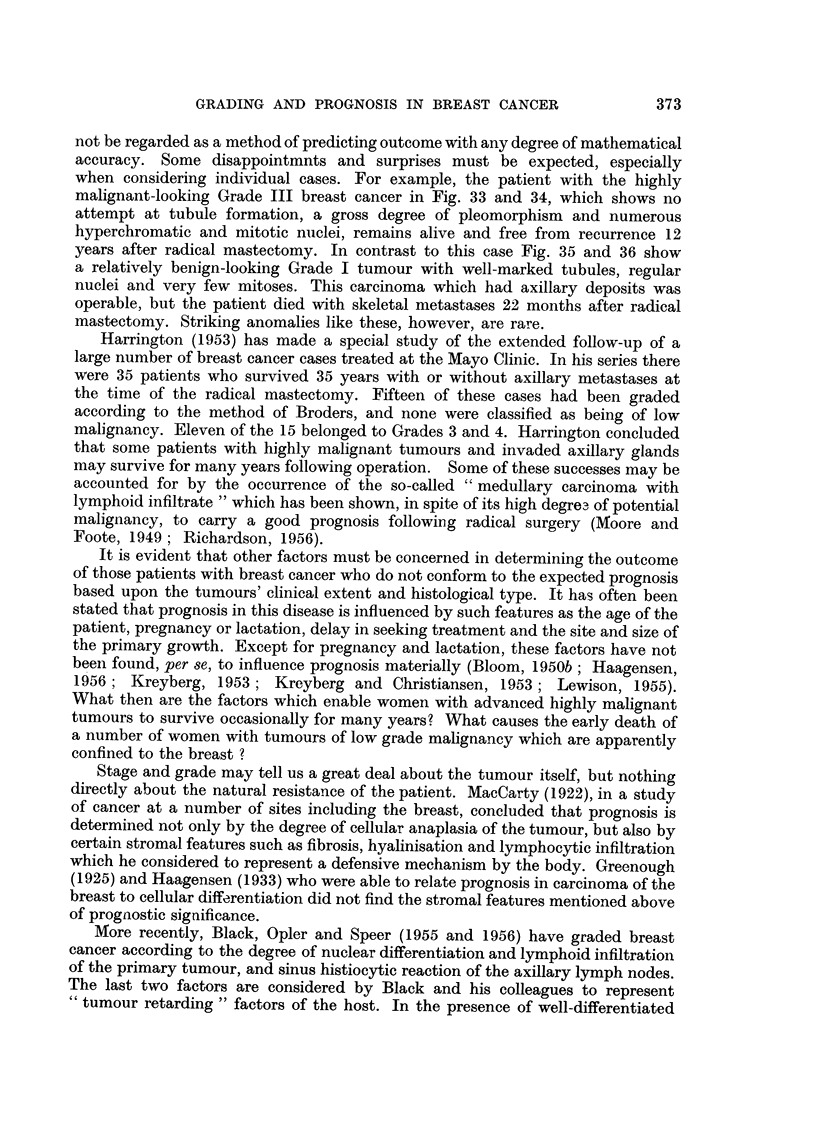

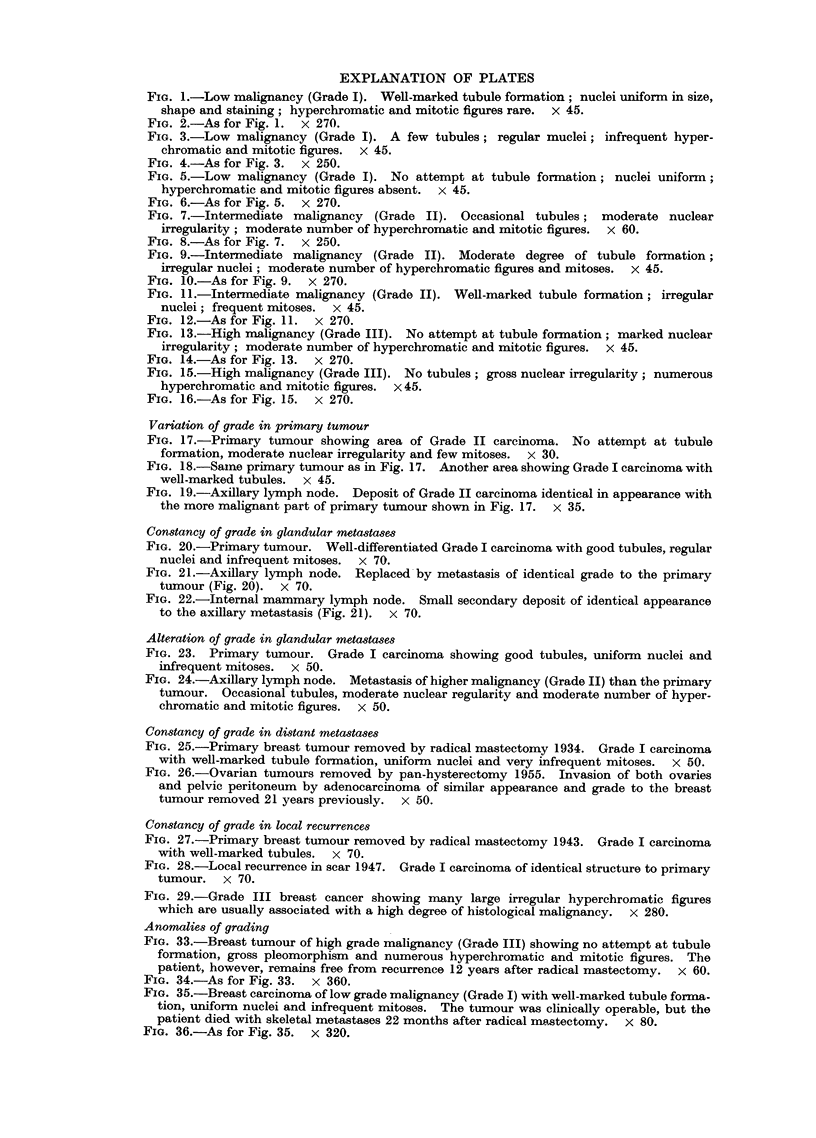

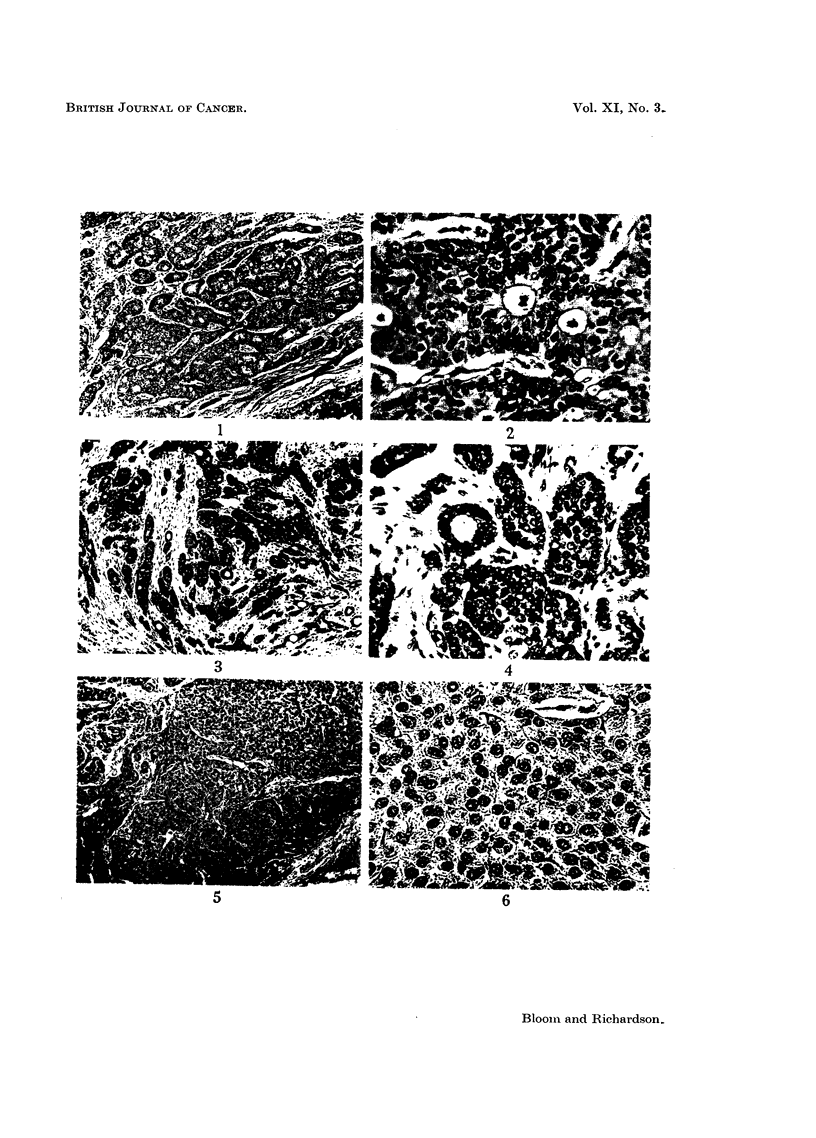

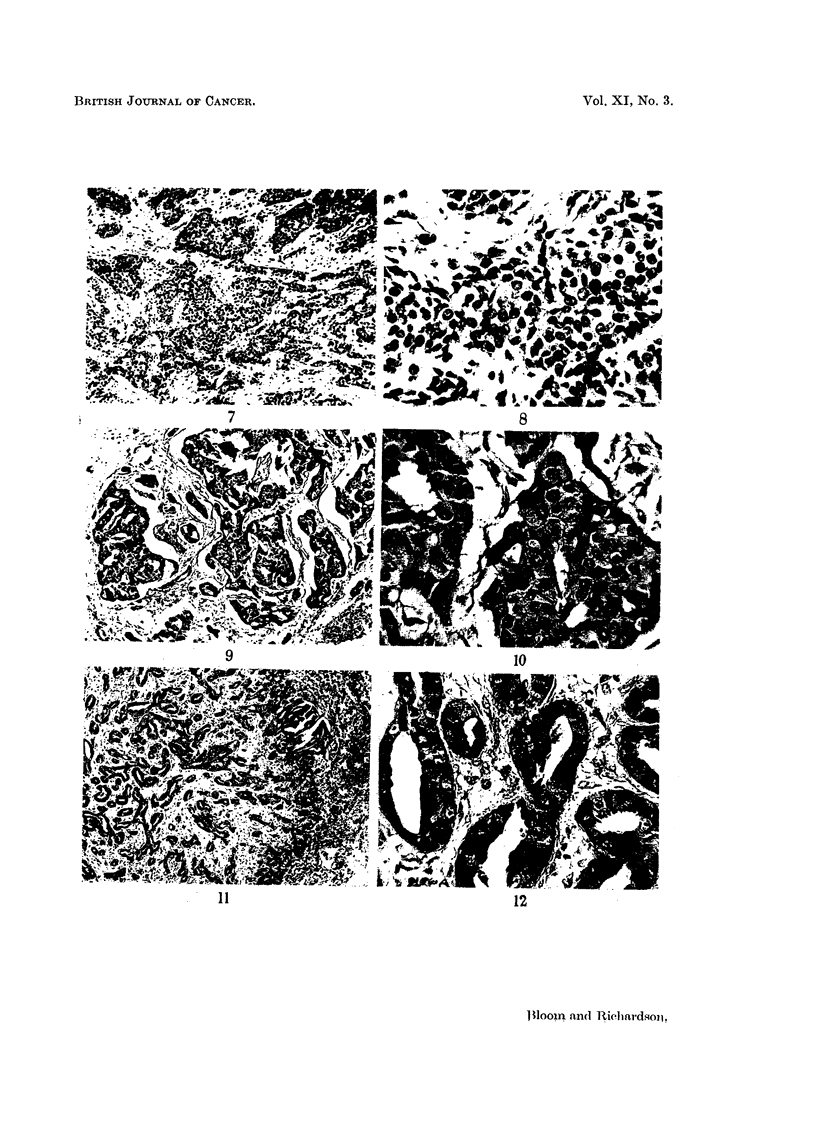

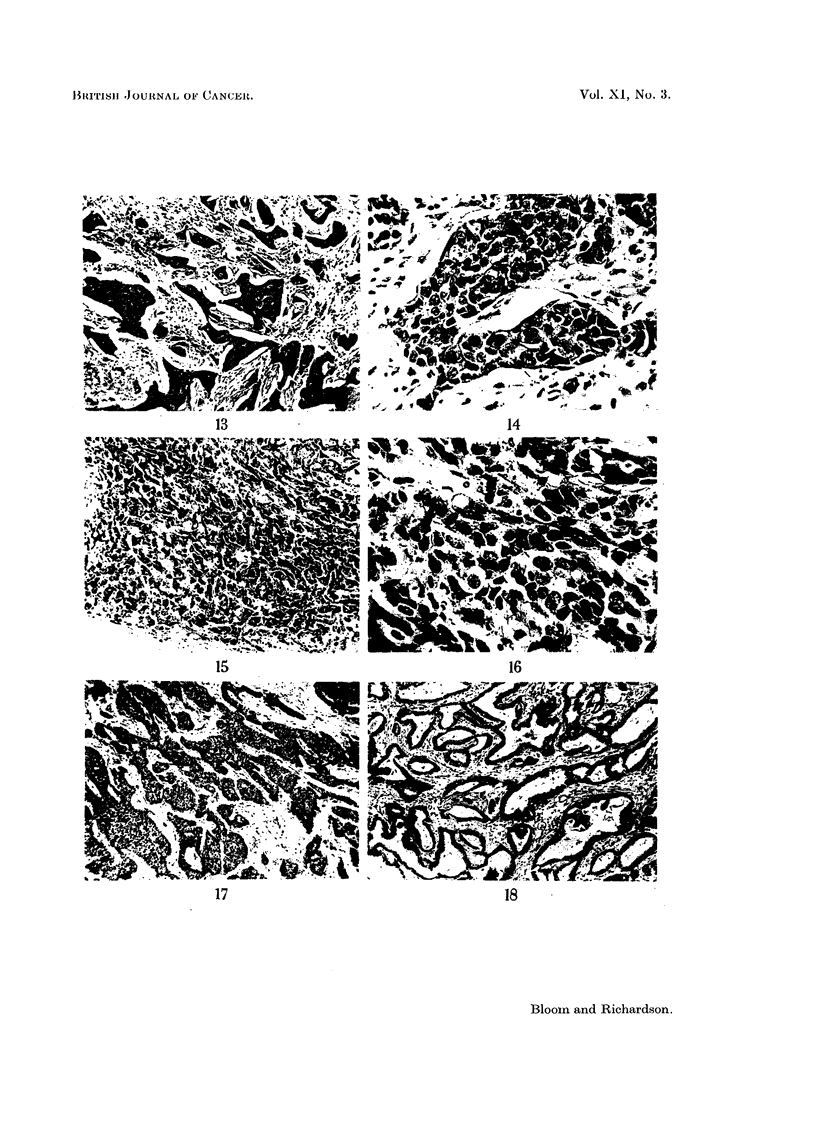

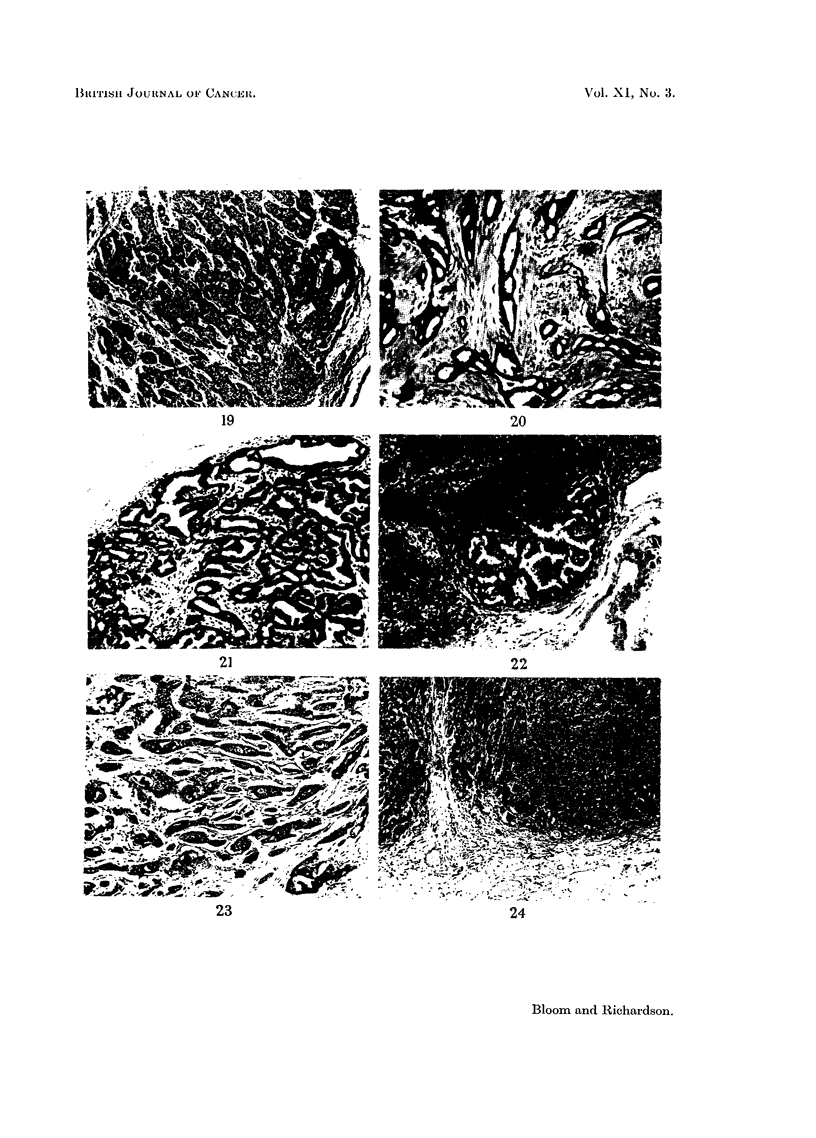

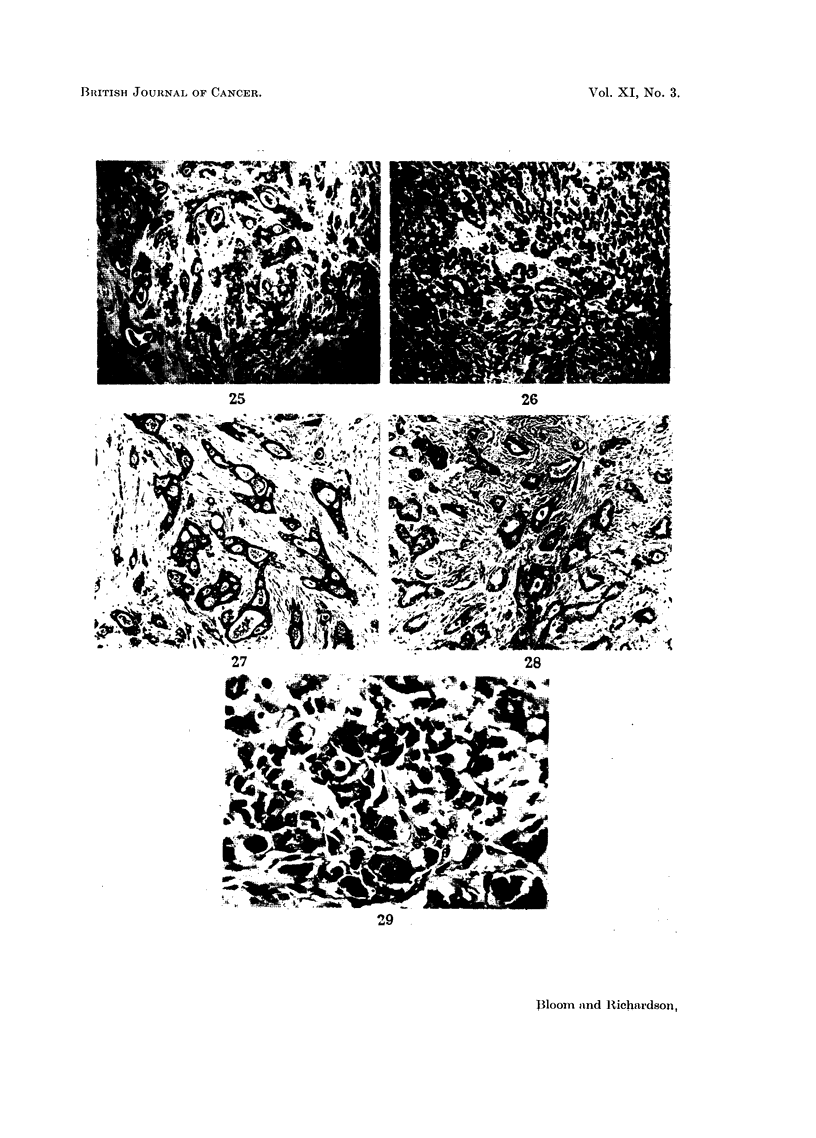

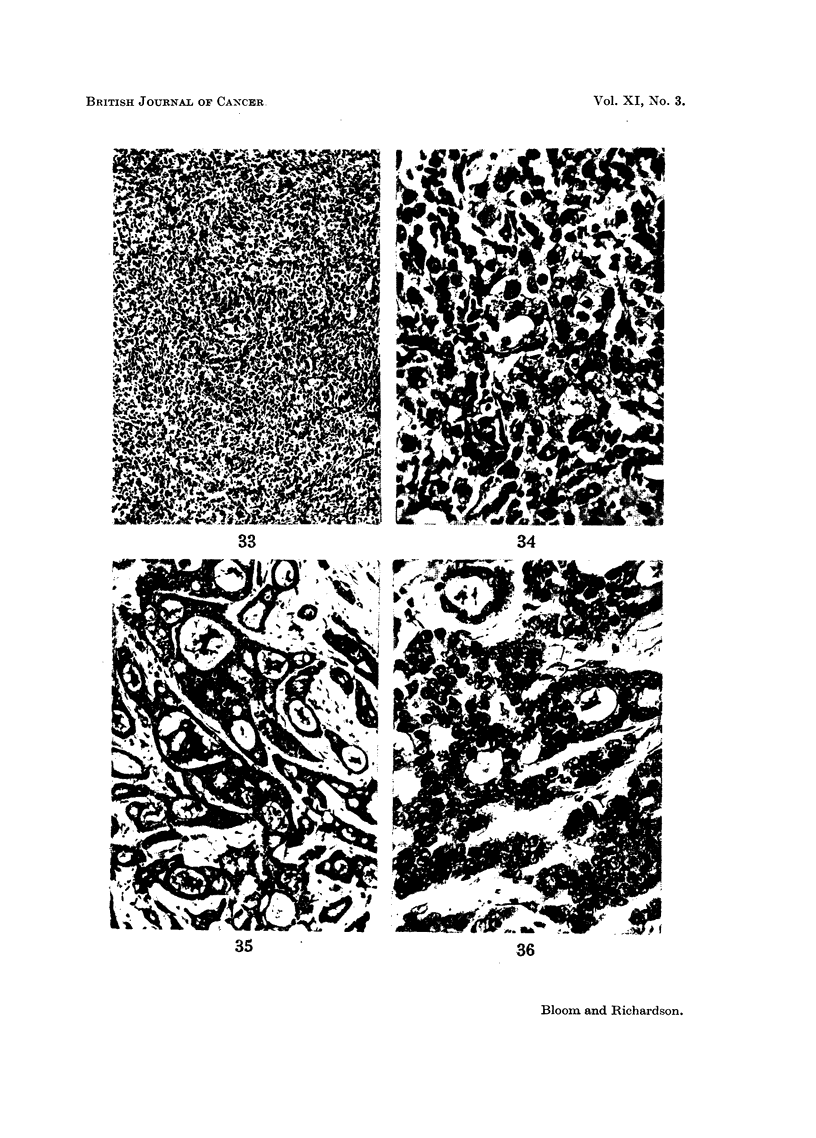

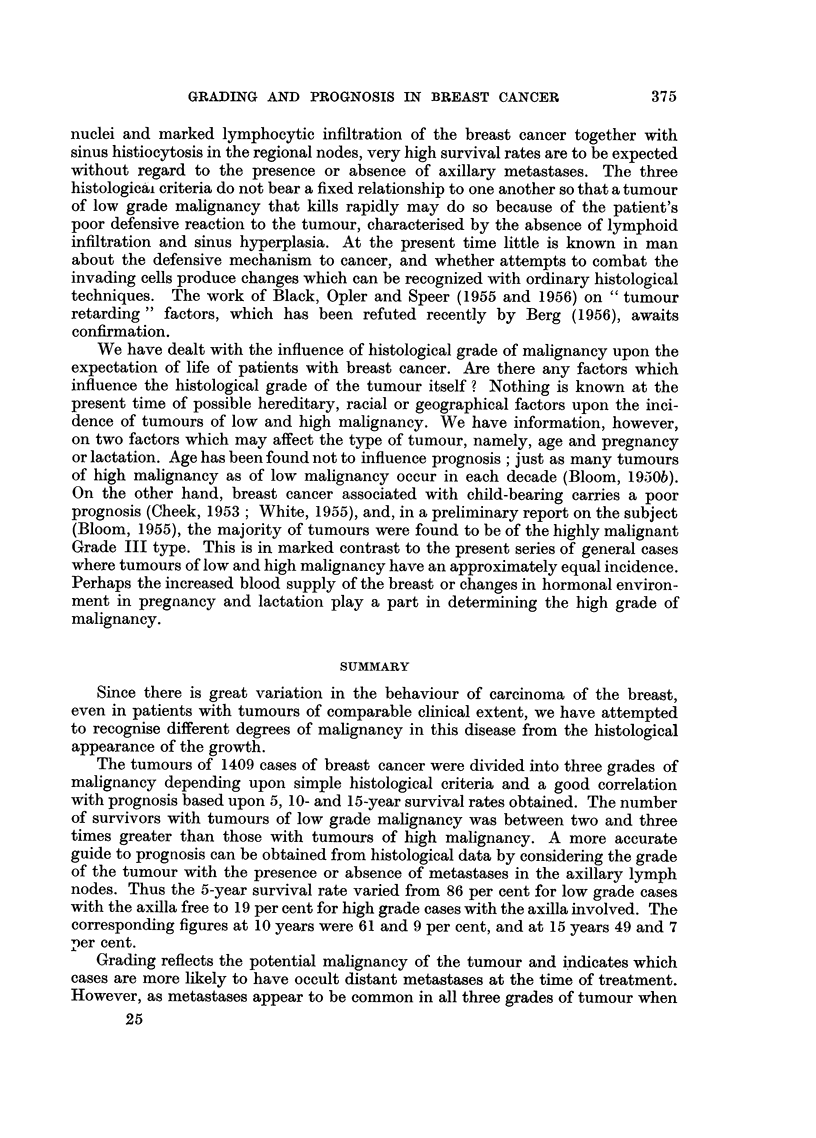

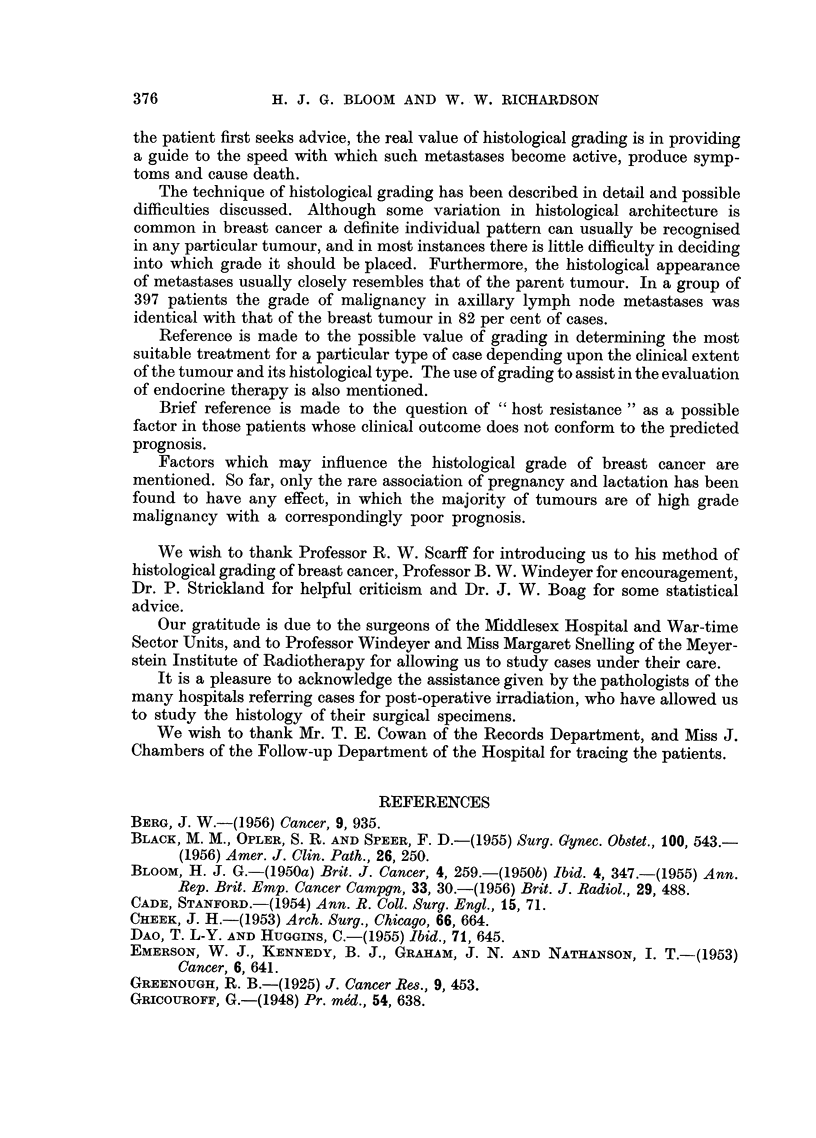

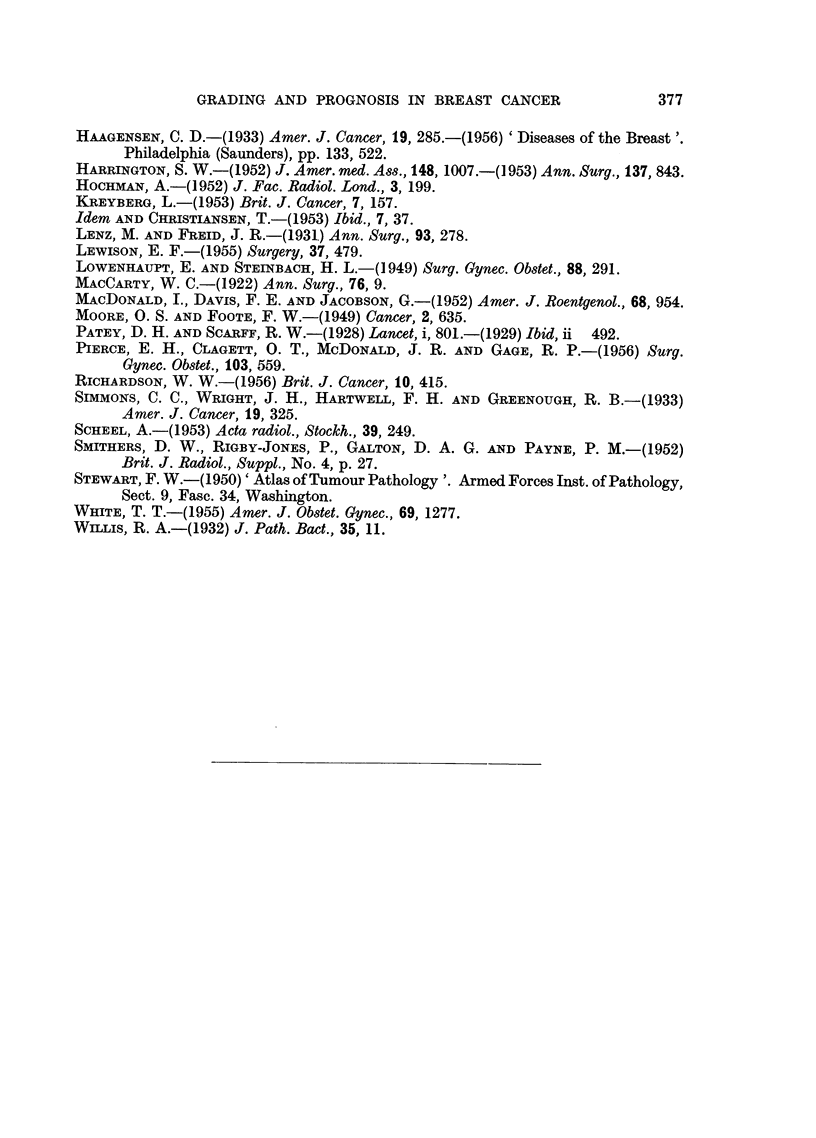


## References

[OCR_01332] BERG J. W. (1956). Sinus histiocytosis: a fallacious measure of host resistance to cancer.. Cancer.

[OCR_01334] BLACK M. M., OPLER S. R., SPEER F. D. (1955). Survival in breast cancer cases in relation to the structure of the primary tumor and regional lymph nodes.. Surg Gynecol Obstet.

[OCR_01376] CLAGETT O. T., GAGE R. P., MCDONALD J. R., PIERCE E. H. (1956). Biopsy of the breast followed by delayed radical mastectomy.. Surg Gynecol Obstet.

[OCR_01348] EMERSON W. J., KENNEDY B. J., GRAHAM J. N., NATHANSON I. T. (1953). Pathology of primary and recurrent carcinoma of the human breast after administration of steroid hormones.. Cancer.

[OCR_01363] KREYBERG L. (1953). The significance of early diagnosis in breast cancer: a study of some common usages of the term.. Br J Cancer.

[OCR_01366] LEWISON E. F. (1955). The problem of prognosis in cancer of the breast.. Surgery.

[OCR_01372] MACDONALD I., DAVIS F. E., JACOBSON G. (1952). Steroid hormone therapy in mammary carcinoma.. Am J Roentgenol Radium Ther Nucl Med.

[OCR_01386] SCHEEL A. (1953). Some prognostic factors, particularly biopsy, in carcinoma of the breast.. Acta radiol.

[OCR_01396] WHITE T. T. (1955). Carcinoma of the breast in the pregnant and the nursing patient; review of 1,375 cases.. Am J Obstet Gynecol.

